# A Theoretical Framework for a Hybrid View of the N400

**DOI:** 10.3389/fpsyg.2021.678020

**Published:** 2021-09-08

**Authors:** Ralf Naumann, Wiebke Petersen

**Affiliations:** Department of Computational Linguistics, Institute for Language and Information Science, Heinrich-Heine-Universität Düsseldorf, Düsseldorf, Germany

**Keywords:** N400, hybrid view, categorization, entropy, predictions, frame theory, probability

## Abstract

In this study, we present a novel theoretical account of the N400 event-related potential (ERP) component. Hybrid views interpret this ERP component in terms of two cognitive operations: (i) access of information, which is related to predictions (predictability component), and (ii) integration of information, which is related to plausibility (plausibility component). Though there is an empirical evidence for this view, what has been left open so far is how these two operations can be defined. In our approach, both components are related to categorization. The critical word and the argument position it is related to are associated with categories that have a graded structure. This graded structure is defined in terms of weights both on attributes and values of features belonging to a category. The weights, in turn, are defined using probability distributions. The predictability component is defined in terms of the information gain with respect to non mismatched features between the two categories. The plausibility component is defined as the difference in the degree of typicality between the two categories. Finally, the N400 amplitude is defined as a function of both components.

## 1. The N400: Functional Characterizations and Empirical Measures

The N400 is a centroparietally negative-going waveform that is largest between 300 and 400 ms after the onset of an incoming word. It was first investigated by Kutas and Hillyard (1980). They found that relative to a coherent control word (e.g., “butter”) a semantic anomalous word (e.g., “socks”) in the final position of the sentence elicited an N400 effect: “He spread the warm bread with butter / socks.” In Kutas and Hillyard (1984), it was observed that the N400 effect does not depend on a semantic violation (see also Hagoort and Brown, 1994). For example, in “Don't touch the wet dog,” the critical word (CW) “dog” elicited a larger N400 amplitude than the CW “paint” in the corresponding sentence “Don't touch the wet paint” though both words satisfy the semantic restrictions imposed by the verb “touch” and the adjective “wet.” Later on, it was investigated how the N400 depends on the wider discourse context. For example, van Berkum et al. (1999) used target sentences like “Jane told the brother that he was exceptionally slow / quick.” If these sentences were embedded in the wider (discourse) context “As agreed upon, Jane was to wake her sister and her brother at five o'clock in the morning. But the sister had already washed herself, and the brother had even got dressed,” the discourse-coherent word “quick” elicited a smaller N400 amplitude than the discourse-anomalous word “slow” in the target sentence. Without this preceding context, this N400 effect was not observed.^1^ Nieuwland and van Berkum (2006) showed that discourse context can overrule lexical properties assigned by a verb to its arguments. The influence of world knowledge in relation to word meanings on the N400 was investigated, e.g., in Hagoort et al. (2004).

Basically, there are two main strands in the debate on the interpretation of the N400 component.^2^ The first one centers on the functional interpretation of this component: does N400 activity correlate with accessing information from semantic memory (access view) or does it correlate with integrating (the representation of) the CW into (the representation of) the preceding context? The most serious problem underlying this debate is that neither “access” nor “integration” has so far been defined in a precise and formal way (for a comprehensive overview see Kuperberg, 2016). For example, “access” has at least been used to refer to (i) lexical access, (ii) semantic access/retrieval, (iii) the effects of lexical prediction on access, and (iv) the effects of semantic prediction on lexical access. How “integration” is interpreted depends, in general, on the underlying theoretical framework. For example, Baggio and Hagoort (2011) use the term to refer to the linguistic operation of unification that combines the linguistic representation of the context with the linguistic representation of the CW. On this view, the N400 correlates with a compositional operation. This is contrasted with an access view according to which retrieving information from semantic memory is a non-compositional operation (see also Lau et al., 2008). Instead, Van Petten and Luka (2012) use the term to simply refer to any effects of context that start to impact as the form features of the incoming word become available (distinguishing this bottom-up primacy from pre-activation). Finally, other approaches, like the computational approach by Rabovsky and McRae (2014), do not assume separate stages for lexical access and subsequent integration.

The second debate centers on which (combinations of) empirical measures underlie N400 activity. Three such measures have been used: predictability, semantic similarity, and plausibility. Predictability of a word is mostly quantized as cloze probability: the percentage of participants in a cloze reading study that used this word to continue a sentence or a text (cloze probability was introduced in Taylor, 1953). Semantic similarity is related to memory-based models of text processing. Such models are based on the assumption that simple lexico-semantic relationships within the internal representation of context interact with lexico-semantic relationships stored in long-term memory and prime upcoming lexical information through spreading activation, called “resonance” (cf. Kuperberg and Jaeger, 2016). On this approach, the context is taken as a bag of words and, therefore, as a lower level representation that is distinct from higher-level representations of the event structure that are based on combinatorial operations, linking the objects (discourse referents), e.g., by thematic roles (“who does what to whom”) (cf. Kuperberg and Jaeger, 2016). Semantic similarity is often quantized by means of latent semantic analysis (LSA, see the articles in Landauer et al., 2007 for details). On this account, pairwise term-to-document semantic similarity values (SSV) are extracted from corpora by calculating the cosine similarities between the vectors corresponding to the critical words and “pseudo-document vectors” that correspond to the prior context up to the critical word (see Kuperberg et al., 2020 for an application). Finally, plausibility can be quantized by offline rating or norming tasks in which participants evaluate the plausibility of the target sentence including the critical word.

A further question that is heavily debated concerns the relation between the functional characterizations (access vs. integration) and the three empirical measures. In this debate too, there is no consensus. For example, some researchers link access to prediction quantized by cloze probabilities (Federmeier and Kutas, 1999; Lau et al., 2008; Kuperberg et al., 2020) while others do not. An example of the latter strategy is the Retrieval-Integration model of Brouwer and colleagues in which access is related to semantic similarity though the similarity is not quantized by LSA (for details see Delogu et al., 2019). Integration is often linked to plausibility. The less plausible the critical word is in relation to its context, the higher is the cost of integrating the word into this context (see e.g., Nieuwland et al., 2019 for discussion). This cost is reflected in the size of the N400 amplitude. However, the correlation between the N400 and predictability and the N400 and plausibility is not necessarily evidence for an access or an integration view, respectively. For example, in the context of “You never forget how to ride a …” “bicycle” is both a more predictable and a more plausible continuation than “elephant” (Nieuwland et al., 2019). The overall greater plausibility of the sentence with the completion “bicycle” can, therefore, also be taken as reflecting facilitated access.^3^

In this study, we will sidestep the issue of how access and integration should or could be defined and the question of how these two theoretical notions can be related to predictability, plausibility, and semantic similarity. The empirical starting point of our account is two important empirical findings about the N400. First, some studies have found that CWs with the same cloze probability differ in N400 activity (see e.g., Federmeier and Kutas, 1999; Kuperberg et al., 2020 and the discussion below in section Predictability, Plausibility, and Semantic Features). Second, there are studies that found a temporal dissociation between a predictability and a plausibility component (in that order) during the N400 time window (see Nieuwland et al., 2019 and section Temporal Dissociations Between Predictability and Plausibility below). Basically, two strategies have been proposed for dealing with these empirical findings. The first strategy takes predictability as central and tries to explain away plausibility by analyzing “same-cloze-different-N400” examples in terms of either differences in the overlap of pre-activated and actually found features (Federmeier and Kutas, 1999) or the number of non-pre-activated features that need to be activated upon encountering the CW (Kuperberg et al., 2020). On the negative side, one has that this strategy fails to give an account of how the plausibility component in the temporal dissociation examples can be reduced to predictability. The second strategy is hybrid views that are mostly based on temporal dissociation examples and in which N400 activity is functionally characterized by both a predictability and a plausibility component (see Nieuwland et al., 2019 and section Temporal Dissociations Between Predictability and Plausibility below). On the negative side, one has that these views do not provide a theoretical model in which predictability and plausibility are given formal definitions except in terms of cloze probability (predictability) and offline ratings (plausibility).

Given these strategies, the two central questions in this debate are whether plausibility can be reduced to predictability and how the temporal dissociation can be accounted for. One strategy for answering this question is to first provide a theoretical model in which both notions are formally defined. Given such a model, one way to proceed is to prove that the definition of plausibility can be reduced to that of predictability and then to show how the relevant data can be accounted for by this definition (reductive strategy). An alternative way is to stay with the two definitions and explain the data in terms of both definitions (hybrid view). In this study, we will adopt the second way. The theoretical model will be based on the notion of a frame (Barsalou, 1992), which is closely related to the notion of a script from cognitive science, Schank and Abelson (1977). The definition of both the predictability and the plausibility component is related to the cognitive operation of categorization.

Similar to prototype theory, we assume that categories have a graded structure. This structure is defined by assigning weights to both attributes and their values. Weights, in turn, are defined by probabilities. This graded structure allows for the definition of typicality, i.e., a binary relation between categories. Having the notion of typicality, it becomes possible to distinguish between information gain and typicality. This can be seen as follows: Given a context built upon the interpretation of the words *w*_1_…*w*_*t*_, a partial representation of a scenario or a script and an event have been construed. For the current event, particular argument positions *arg*, are still open in the sense that none of the words *w*_*i*_ are assigned to this position. With each *arg* a category *C*_*arg*_ is associated. If a CW *w*_*CW*_ is encountered that fills the open argument position *arg*, *arg* is discharged. The word *w*_*CW*_ expresses a category *C*_*CW*_. The found category *C*_*CW*_ must be combined with the categorical information *C*_*arg*_ required by the event. This combination will be modeled as an update operation: *C*_*arg*_ is updated with *C*_*CW*_. This update operation is the composition of two operations that are related to categorization in the following way. The first operation determines the information gain that is got by *C*_*arg*_ given *C*_*CW*_ by computing the features in *C*_*arg*_ that are not disconfirmed by *C*_*CW*_. This operation is related to predictability: which information in *C*_*arg*_ is retained after the combination of *C*_*arg*_ and *C*_*CW*_? The second operation computes the typicality of *C*_*CW*_ relative to *C*_*arg*_. This computation correlates with plausibility because typicality can be taken as answering the question of how plausible are the features in *C*_*CW*_ relative to those in *C*_*arg*_. Hence, whereas predictability focuses on *C*_*arg*_ (which features in this category are not disconfirmed?), plausibility focuses on *C*_*CW*_ (how typical are the features in this category in relation to *C*_*arg*_?).

The reminder of this study is organized as follows. In section 1.1 we discuss feature-based approaches with special attention to studies focusing on animacy and the question whether plausibility can be reduced to predictability. In section 1.2, we discuss studies that found a temporal dissociation between predictability and plausibility during the N400 time window. The topic of 1.3 is the question whether plausibility in the N400 time window refers to whole event structures or to concepts related to objects participating in such structures. In section 2, we define our hybrid view in an informal manner by relating plausibility to typicality and by relating predictability to information gain. Finally, in section 3 an outline of the formal framework is presented together with a discussion of some relevant examples from the previous sections in this framework. This section closes with the sketch of an extension of the framework to script knowledge.

### 1.1. Predictability, Plausibility, and Semantic Features

As mentioned in the introduction, the most prominent way of operationalizing predictability is by cloze probability. The correlation between word predictability so defined and the amplitude of the N400 is well established with correlations of *r* = 0.8 or even higher for some studies (for details see Nieuwland et al., 2019).

One kind of counterexample to this dependency is cases in which two CWs with the same low cloze probability elicit N400 amplitudes of different size. For example, Federmeier and Kutas (1999) compared BC (best completions, i.e., highest cloze probability) with two other types of completions: those that came from the same semantic category as the best completion (within-category violations) and those that did not (between-category violations).

**Table:** 

(1)	They wanted to make the hotel look more like a tropical resort. So along the driveway they planted rows of palms / pines / tulips.

Though both “pines” and “tulips” in (1) have the same low cloze probability, the N400 amplitude for “pines” is smaller than that for “tulips.” Federmeier and Kutas explain this pattern by assuming that semantic memory has a categorical structure such that categories are represented by interrelated sets of features instead of atomic units. Objects belonging to the same category share, in general, many features, namely those that are common to all members of the category. Given a particular context, specific features of a category are pre-activated. The greater the overlap between these pre-activated features and the features associated with the category expressed by the CW, the more the N400 amplitude is attenuated. For example, the context prior to the CW in (1) pre-activates features like “habitat = tropics” and “height = tall.” The best completion “palms” satisfies all these pre-activated features. Though “pines” fails to satisfy “habitat = tropics,” it satisfies “height = tall” and all features of the category “tree,” which is the category of the BC “palms.” Hence, “pines” is a within-category violation. By contrast, “tulips” is a between-category violation because tulips are flowers and not trees although there is a common supercategory, namely “plant.” Hence, “tulips” does not satisfy the features that are specific of trees, and in addition, it fails to satisfy “habitat = tropics” and “height = tall.”

A second kind of studies in which the N400 amplitude differed despite identical (low) cloze probabilities involves animacy violations. One example is the study by Kuperberg et al. (2020). They used examples like those in (2) where contexts where either categorized as high constraint (HC) as in (2-a) or as low constraint (LC) as in (2-b).

**Table:** 

(2)	a.	The lifeguards received a report of sharks right near the beach. Their immediate concern was to prevent any incidents in the sea. Hence, they cautioned the swimmers / trainees / drawer ….
	b.	Eric and Grant received the news late in the day. They mulled over the information and decided it was better to act sooner rather than later. Hence, they cautioned the trainees / drawer ….

In the four conditions (HC vs. LC and “trainees” vs. “drawer”) predictability quantized by cloze and semantic similarity quantized by LSA were held constant. The authors found that the N400 amplitude for “trainees” as well as that of “drawer” were independent of whether the context described a HC or a LC scenario. However, “drawer” elicited a slightly, but significantly, larger N400 amplitude than “trainees” (in both scenarios).

The authors interpret N400 activity as reflecting access to the semantic features associated with new bottom-up input that has not already been predicted (Kuperberg et al., 2020, p. 3). For example, in the LC scenario (2-b) only features that are characteristic of animate objects like sentient and can_move are pre-activated. By contrast, in the HC scenario (2-a) additional features like in_water and afloat are pre-activated. The CW “swimmers” in the HC scenario satisfies all of these features so that no new semantic information needs to be activated. As a result, “swimmers” only elicits a small N400 amplitude. The CW “trainees” satisfies the features related to animacy: sentient and can_move in both scenarios. However, in the HC scenario it fails to satisfy the additional features imposed by the context. Hence, a comprehender must retrieve additional features that more specifically characterize trainees like learning and novice in that context. Since more features need to be activated, the amplitude of the N400 for “trainees” is larger than that for “swimmers.” Finally, in both scenarios “drawer” matches none of the pre-activated features. Therefore, a comprehender must retrieve all of its properties including features like storage and can_open. Hence, the N400 amplitude for “drawer” is the largest. The example provides evidence that even if the context is low-constraining the verb can already activate features of the upcoming word that are related to animacy (compare LC scenario). By contrast, the activation of other features depends on other factors like contextual information and context strength.

Empirical evidence for this distinguished role of animacy features comes from the study Wang et al. (2020). The authors exploited the inherent difference in the semantic similarity structure of animate and inanimate nouns. Objects denoted by animate nouns share more co-occurring features than objects denoted by inanimate nouns. This difference shows up in the fact that the category “inanimate” has a larger number of subcategories than the category “animate.” In the brain, semantic features are thought to be represented within widely distributed networks (see, for example, Huth et al., 2016). These differences in the way features are stored can give rise to differences in similarity among the spatial patterns of neural activity associated with the processing of words that are related to these categories (Wang et al., 2020). The authors used representational similarity analysis (RSA) (Kriegeskorte et al., 2008), which is one way of detecting such neural differences, in combination with magnetoencephalography and electroencephalography (MEG and EEG). Their hypotheses were as follows: (i) If comprehenders can use the animacy constraints of verbs to predict the semantic features associated with the animacy of an upcoming noun, then the similarity in spatial patterns should be greater following animate-constraining than inanimate-constraining verbs; (ii) if these animacy predictions are generated regardless of being able to predict specific words, this effect should be independent of context strength, i.e., it should be the same in HC and in LC scenarios. They used three sentence scenarios like those in (2) and (3).

**Table:** 

(3)	a.	Judith was working on the origami project for her office fundraiser. She was starting to get frustrated because it was her third attempt at making a crane. Nevertheless, she unfolded the … HC
	b.	Judith was nearing the end of her rope. She didn't think she could keep going. Nevertheless, she unfolded the … LC

Verbs in the final sentences constrained for either an animate [e.g., (2)] or an inanimate theme [e.g., (3)] and the broader discourse constrained for either a specific noun (HC scenario) or multiple nouns belonging to the same animacy category (LC scenario). The authors found that the spatial pattern of neural activity for animate-constraining verbs was significantly more similar than for inanimate-constraining verbs in both datasets (MEG/EEG). Furthermore, this effect was independent of context strength: It was just as large following HC as following LC scenarios. This effect began after the peak of the N400 component evoked by the verb and, therefore, past the stage at which comprehenders are likely to have accessed the lexico-semantic features of the verb and well before the direct object (theme) was actually encountered.^4^

Given examples like (2) and the results of Wang et al. (2020), the following hypothesis can be put forth.

**Table:** 

(4)	If two CWs *w*_1_ and *w*_2_ have the same low cloze probability and *w*_1_ satisfies the animacy constraints imposed by the verb whereas *w*_2_ does not, the N400 amplitude of *w*_2_ is larger than that of *w*_1_.

Though this hypothesis may seem to be unrelated to the account of Federmeier and Kutas, there is the following relationship. Consider (2-a). Both “swimmers” and “trainees” denote animate objects whereas “drawer” denotes inanimate ones. Hence, “swimmers” and “trainees” share a common supercategory, “animate,” whereas “drawer” is a between-category violation because the common supercategory is “material object.” The difference between the two examples is the level in the categorical hierarchy at which (dis-)similarities are located. Whereas, in (1) this is a very concrete level (“tree,” “flower,” and “plant”), and it is a more abstract level in (2). On this modeling, “trainees” shares with the set of pre-activated features (or the best completion “swimmers”) the animacy features that are specific to all objects falling under the corresponding category, whereas this does not hold for “drawer.” Since the activation of animacy features is independent of context strength, this argument applies mutatis mutandis also to the LC scenario in (2-b). Further evidence for the distinguished role of the animacy features comes from Paczynski and Kuperberg (2011). For example, the authors used examples like those in (5).

**Table:** 

(5)	a.	At headquarters the manager interviewed the applicant for thirty minutes.
	b.	At headquarters the manager surprised the applicant after thirty minutes.
	c.	At headquarters the manager interviewed the application for thirty minutes.
	d.	At headquarters the manager surprised the application after thirty minutes.

The CW in the violating conditions differs from the CW in the non-violating conditions primarily in its animacy features. Furthermore, all CWs had the same low cloze probability. The authors found that the N400 amplitude in the non-violating cases (5-a) and (5-b) did not differ, i.e., it was not modulated by the thematic role (experiencer for “surprise” vs. patient for “interview”). Similarly, the N400 amplitude showed no difference in the two violating conditions though it was larger than in the non-violating conditions.

However, there are a number of studies that provide counterexamples to the claim that animacy features play the role attributed to them in hypothesis (4). The study Szewczyk and Schriefers (2011) shows that this need not be the case. The target language used in this study was Polish. The authors used scenarios in which the target sentence had a canonical subject, verb, object (SVO) order and in which subjects were unambiguously marked by nominative case and direct objects were unambiguously marked by accusative case. In all examples, either an animate or an inanimate object was highly expected. In one condition, the direct object satisfied all constraints, i.e., all selection restrictions imposed by the verb and contextual constraints. In a second condition the (in-)animacy constraint was violated and in a third condition, the (in-)animacy constraint was satisfied, but either another selection restriction or a contextual constraint was violated. Both violation conditions had a cloze probability of 0, whereas cloze probability was 0.44 in the non-violating condition. Below the English translation of two examples used in the study is given.^5^

**Table:** 

(6)	a.	Although it was late autumn and bitter cold, little John was running in the backyard with his neck bare. His worried grandma prepared some wool and knitted a scarf (nv) / a medicine (sv) / an employee (av)
	b.	A young RAF pilot was returning to his base when he suddenly notices a Messerschmitt. The pilot fought a duel shooting down the airplane (nv) / the scarf (sv) / the patient (av).

The authors found that both kinds of violations elicited an N400 effect relative to the non-violating condition. Most importantly, the N400 amplitudes did not differ, i.e., both kinds of violations elicited an amplitude of the same size. If animacy violations were worse than others, than “medicine” in (6-a) should elicit a smaller N400 amplitude than “employee” because it satisfies the (in-)animacy constraints whereas “employee” does not. Following the same argument “scarf” in (6-b) should elicit a smaller N400 than “patient.” Similar results have been reported in Quante et al. (2018). Two examples from this study are given in (7).

**Table:** 

(7)	a.	Peter stand bei Morgendämmerung auf, fuhr den ganzen Tag Traktor und fütterte abends seine Kühe. An manchen Tagen wäre er aber lieber kein Bauer / Trick sondern ein unbekümmertes Kind.Peter gets up at dawn, drives the tractor all day and feeds his cows in the evening. On somw days he would rather not be a farmer / trick but a carefree child.
	b.	Luisas neues WG-Zimmer war sehr klein, hatte aber hohe Decken. Um Platz zu sparen, kaufte sie sich deshalb ein Hochbett / Schwein im Baumarkt.Luisa's new room was very small but had high ceiling. To save space, she bought herself a loft bed / pig in the store.

Though “Trick” violates the animacy constraint while “Schwein” does not (pigs can be bought), there was no difference in the N400 amplitude between the two conditions.

What these counterexamples show is that violations of constraints that are not related to animacy violations can have the same effect on N400 activity: An N400 amplitude of the same size is elicited. This provides evidence against the hypothesis in (4). More generally, one has the following. At least implicitly, the hypothesis (4) is based on the following assumption. Features are related to a particular level in a categorical hierarchy. The higher this level, the greater is the set of violated features and the higher is the corresponding N400 amplitude. For example, animacy features are related to the distinction at the (high) level of material objects. A violation of an animacy feature results in a violation of many features and a pronounced N400. Other types of features are related to lower levels in the hierarchy. Two types of such features related to N400 activity that need to be distinguished are (a) selection restrictions imposed by the verb that are not related to animacy and (b) features that are imposed by the context. As an example of the former type, consider the verb “caution.” It requires its theme argument to be in danger. The verb “knit” imposes the constraint that its theme argument can be manufactured by this type of action. These constraints are lower in the hierarchy because they can be failed to be satisfied while the animacy features are still satisfied. For example, in (6-a) “medicine” is inanimate but it cannot be manufactured by a knitting process. Constraints imposed by the context are even lower in the hierarchy. For example in (2-a), the objects cautioned are most likely persons (and, therefore, human) who happen to be afloat and in water. What the counterexamples discussed above show on this modeling is that at least in particular contexts the failure to satisfy a particular feature that is not related to an animacy violation and that is therefore lower in the categorical hierarchy can have the same effect on N400 activity as a violation of an animacy constraint, contrary to the hypothesis in (4). More importantly, these counterexample provide evidence that differences in N400 activity for CWs with the same low cloze probability can be explained solely in terms of differences at the level of predictability. For example as discussed above, Kuperberg et al. (2020) explicitly adopt the strategy that the difference between “trainees” and “drawer” is a difference in pre-activated and features activated upon encountering the CW and not as a difference in plausibility over and above predictability. As a result, plausibility is “explained away” in favor of predictability. What is left open by these counterexamples is, of course, whether this additional component in N400 activity is in the effect plausibility of an event structure (or a sentence). Before discussing this question, we will discuss a second problem for strategies that are based on “explaining away” plausibility.

Critical words can be preceded by prenominal elements like determiners and adjectives that provide information about the category expressed by this CW. These prenominal elements can either confirm pre-activated features in the preceding context (matching condition) or not (mismatching condition). If N400 activity can be characterized solely in terms of the size of the set of correctly pre-activated features, the question arises on how the effect of mismatching features can be explained in this approach. Before tackling this question, we will present the results of the study Boudewyn et al. (2015) that examined the influence of prenominal adjectives on N400 activity. More specifically, the authors investigated the pre-activation of features by the ERP response to adjectives that are not themselves predictable but denote features of objects that are denoted by highly predictable not yet presented nouns. To this end, they constructed two-sentence stories in which a noun in the second (target) sentence was highly predictable (e.g., “cake”) and was preceded by an adjective that denotes either a typical or atypical feature of objects denoted by the critical noun. An example story is given in (8).

**Table:** 

(8)	Frank was throwing a birthday party, and he had made the dessert from scratch. After everyone sang, he sliced up some sweet/healthy and tasty cake/veggies that looked delicious.

Event-related potentials were examined at two points during the second sentence. The first time lock was to the unpredictable adjective and the second time lock was to the critical noun. For the noun, there were four different conditions: (i) locally consistent and globally predictable noun (“sweet and tasty cake”), (ii) locally inconsistent and globally predictable noun (“healthy and tasty cake”), (iii) locally consistent and globally unpredictable noun (“healthy and tasty veggies”), and (iv) locally inconsistent and globally unpredictable noun (“sweet and tasty veggies”). Predictability of the noun was established by a cloze test (cloze for BC : 78% and 0% for non-BC). All adjectives were unexpected, regardless of whether norming participants were asked to provide a single-word continuation (cloze : 0.01%) or a multiple-word continuation (cloze : 1.81%).

For the adjectives, the authors found a reduced N400 amplitude for adjectives denoting features consistent with the best completion compared to adjectives denoting inconsistent ones. The authors conclude that semantic features of objects denoted by highly predictable nouns are accessible before the predictable noun is encountered. At the critical noun, they found a graded effect of global predictability and local consistency, with (i) the smallest N400 amplitude to globally predictable, locally consistent nouns (“sweet and tasty cake”), followed by globally predictable, locally inconsistent nouns (“healthy and tasty cake”) with a slightly, but significantly, larger amplitude than for “sweet and tasty cake.” then follows the globally unpredictable, locally consistent nouns (“healthy and tasty veggies”) and finally one has the globally unpredictable and locally inconsistent nouns (“sweet and tasty veggies”).

Consider first the N400 at the (mismatching) prenominal element. Before the prenominal element is encountered, the context raises expectations about the theme of “slice up.” For example, it can be sliced, served as a dessert, and served at a birthday party. Hence, features that are typical of cake-like “sweet” are pre-activated. What happens if “healthy” is encountered instead? This feature applies to different sorts of food that can be served and sliced up. However, in general, “healthy” is not a defining property of a category in the sense that it either applies to all exemplars belonging to the category or to none. Thus, the question arises whether “healthy” is a feature of cake or veggies or not. If one assumes that it is a features of the latter (because veggies are normally healthy) but not of the former (because the cake is rarely healthy), “healthy” contributes to the feature overlap if the CW is “veggies” but not if the CW is “cake.” A second problem is related to correlations between features. For example, “healthy” correlates with “sweet.” Knowing that some food is “healthy” will, in general, lower the expectation that it is in addition sweet because healthy food is, in general, not sweet. Applied to (8), one has the following: encountering “healthy” will lower the expectation for “sweet,” which is a consequence of the fact that predicting is, in general, a non-monotonic process.^6^ Does this have the effect that “sweet” no longer belongs to the set of pre-activated features? If the answer is “yes,” this suggests that “veggies” is more expected because veggies are more likely to be healthy and not sweet (= both features are an element of the feature overlap) whereas cake is more likely to be sweet and not healthy (= both features don't belong to the feature overlap). As a result, “veggies” is more likely to be the CW than “cake” so that the N400 amplitude elicited by the former should be smaller than that elicited by the latter. However, this is not compatible with the results of Boudewyn et al.

The above discussion calls into question the assumption that N400 activity can be characterized by a single operation on features based solely on criteria like “confirmed” (or “matched”) vs. “disconfirmed” (or “mismatched”). What seems to be missing is the possibility of expressing the condition that a confirmed or disconfirmed feature is, in addition, a feature that normally or typically belongs (or does not belong) to a category like this that is the case for “healthy” and “sweet” in relation to cake and veggies. The reason for this is that categories are not defined in terms of definitional properties, i.e., a particular set of features that together provide necessary and sufficient conditions for membership in this category. Rather, categories are defined as graded structures that allow for the definition of typicality (see e.g., Rosch and Mervis, 1975). If viewed from the debate on predictability vs. plausibility, the above discussion can be interpreted in the following way. The relation between N400 activity and prenominal elements suggests that in addition to confirmed vs. disconfirmed the distinction between typical vs. non-typical plays a role for N400 activity. If one correlates “confirmed/disconfirmed” with predictability, “typical/non-typical” is correlated with plausibility using the results from above on the role of animacy features. When taken together, the discussion in this section suggests the following three hypotheses related to N400 activity.

HT1: Categories have a graded (or prototypical) structure which allows for distinguishing between typical and atypical features (e.g., sweet vs. healthy for cake).

How should typicality be defined? One ingredient (component) is the (subjective) probabilities of a comprehender that a category has a particular feature. These probabilities are based on both world and linguistic knowledge. For example, the probability that veggies are healthy is higher than that for cakes, whereas for the feature “sweet” the opposite holds. A second ingredient is the relevance (weight and diagnosticity) of an attribute in a particular context. For example, in (7-b) the buying is carried out with the particular goal to save space. Any objects that are not conducive reaching this goal are excluded on this occasion, independently of whether they satisfy the selection restrictions imposed by the verb. Hence, features related to the goal of saving space are more relevant than other features though they also hold of the object, e.g., inanimacy features in (7-b). Relevance need not be related to a goal. In scenario (6-a), attributes related to the way the object is manufactured are more relevant than other attributes related to inanimacy.

HT2: The graded structure of categories is context-dependent. The context-dependency shows up in weights on attributes. Typicality is defined in terms of weights on attributes and weights on values.

Typicality defined in terms of weights on attributes and weights on values must be distinguished from (correct) predictions. Consider again the scenario of the birthday party. Upon encountering the prenominal element “healthy,” the corresponding feature becomes pre-activated. It provides evidence for “veggies” and evidence against “cake.” However, this evidence can be counterbalanced by typicality. In this particular context, the feature “healthy” has a low relevance (weight) because other features like “sweet” and “served_at_a_birthday-party” are more relevant. This has the effect that the overall contribution of this pre-activated feature to N400 activity is lower than that of a pre-activated feature with higher relevance. As a result, one has that the contribution of a pre-activated feature to N400 activity cannot be reduced to a difference in confirmed or disconfirmed prediction (“healthy” is confirmed by “veggies” but disconfirmed by “cake”). Rather, it also matters how typical these features are relative to the pre-activated features. Hence, two CWs may not differ with respect to prediction “accuracy” though they differ with respect to how typical they are relative to the set of pre-activated features. When taken together, we get the following further hypothesis.

HT3: The contribution of a feature to N400 activity is a function of both its pre-activation and its typicality.

According to the above three hypotheses, differences in N400 amplitude are not reduced to differences in pre-activated features but in addition also reflect differences in the graded structure of categories. Hence, plausibility is not “explained away” as in the approaches by Federmeier and Kutas and that of Kuperberg and colleagues. Two principle assumptions of an account based on the three hypotheses above are as follows: (i) N400 activity is correlated to two different components: information gain (prediction) and (context-sensitive) typicality and (ii) plausibility is, in effect, typicality between two concepts and not the plausibility of an event structure (or of a sentence). In the next two sections, we will review evidence for these two assumptions.

### 1.2. Temporal Dissociations Between Predictability and Plausibility

The so-called hybrid views (see Nieuwland et al., 2019, and references cited therein) claim that N400 activity does not index a single process but a cascade of semantic activation and integration processes. Whereas, the (non-compositional) activation component is correlated to predictability, the (compositional) integration component is correlated to plausibility. Furthermore, effects of predictability and plausibility can both be observed in the N400 time window, but effects of predictability precede and may even be functionally distinct from those of plausibility (Nieuwland et al., 2019). The more general point of these approaches is a functional interpretation of ERP components according to which they most likely reflect “the combined activity of multiple subcomponents that are associated with related yet distinct cognitive processes” (Nieuwland et al., 2019, p. 20).

The main empirical evidence for the hybrid view comes from studies in which predictability and plausibility are independently varied, and a temporal dissociation between effects of these two factors is observed in the N400 time window. Lau et al. (2016) examined modulations of the N400 amplitude associated with independent manipulations of predictability. They used an adjective-noun paradigm that allowed for contrasting the effects of contextual predictability and semantic plausibility on the N400 amplitude by holding one of the two factors constant. In particular, they compared implausible adjective-noun combinations to plausible adjective-noun combinations in which the predictability of the noun given the adjective was very low (*p* < 0.005). To create balanced plausible and implausible sets, they crossed animate nouns and inanimate nouns with adjectives that must modify animate nouns and with adjectives that usually modify inanimate nouns. Example combinations are given in Table 1.

**Table 1:** Example items in the Lau et al. study.

Predictability manipulation	Plausible predictable runny nose mashed potato	Plausible unpredictable dainty nose shredded potato
Plausibility manipulation	Plausible unpredictable yellow bag healthy cat	Implausible unpredictable innocent bag empty cat

Predictability was computed using corpus counts instead of cloze probabilities. Plausibility was computed in an offline rating study using a scale from 1 to 7 according to what degree the adjective-noun combination made sense. Plausible items were rated much higher than implausible ones (mean: 6.59 vs. 1.75). The authors found a large effect of predictability (runny nose vs. dainty nose) with a central posterior distribution and a small effect of plausibility (yellow bag vs. innocent bag) with a leftward distribution. Furthermore, they observed a temporal dissociation of the two effects. Whereas the predictability effect appeared to onset by around 200 ms, the N400 difference due to implausibility appeared to onset substantially later.

A second study is that by Brothers et al. (2015). They used moderately constraining (cloze BC : 50%) two-sentence passages like the following.

**Table:** 

(9)	The author was writing another chapter about the fictional detective. To date, he thinks it will be his most popular novel / book.

The context before the critical word was constructed in such a way to moderately constrain toward two alternative completions that were equally likely given this preceding context, e.g., “novel” and “book” in the above example. The second set of passages was moderately constraining toward an unrelated target, e.g., “dish,” but formed a low-cloze context for the actual final word, e.g., “novel,” that was unpredictable (cloze : <1%), though semantically coherent.

**Table:** 

(10)	Everyone congratulated the chef on all his hard work. To date, he thinks it will be his most popular dish / novel.

Participants were instructed to actively predict the final word of each passage and to respond after each trial whether their prediction was correct. By separately averaging ERP trials for predicted (“novel”) and unpredicted (“book”) targets in the first passage, the authors isolated processing differences at the final CW that were uniquely driven by prediction accuracy [prediction effect (accuracy)]. The second, control, passage was used to compare unpredicted target words in the first passage (predicted: book, found: novel) with unpredicted targets in low-cloze contexts (predicted: dish, found : novel). Any differential activity between these two conditions should index the amount of semantic or discourse-level facilitation provided by the preceding context (contextual support).

For the N400 amplitude, the authors found that predicted CWs had the smallest amplitude, followed by unpredicted CWs in medium-cloze contexts, and finally CWs in low-cloze contexts. Most importantly, there was a strong temporal dissociation between effects of prediction and context facilitation. In the N400 time window, the peak of the prediction effect occurred earlier (380 ms) than that of the context effect (around 480 ms). The authors used a multiple regression analysis to single out which factors of the context were responsible for the context effect. Possible candidates were as follows: plausibility, semantic similarity, and semantic feature overlap. Plausibility was computed using offline plausibility ratings. Semantic similarity was calculated using LSA. For semantic feature overlap, the authors used first the results of the cloze norming procedure to determine the most likely completions of each low-cloze passage and the next best completion of each medium-cloze passage. They then used LSA to compute the degree of semantic overlap between each alternate completion and the actual final word, e.g., book-novel = 0.50 and dish-novel = 0.04. The result of this regression analysis showed that the N400 amplitude approximately 100 ms after the onset of the prediction effect was strongly correlated with (i) the degree of shared semantic overlap between the CW and the next best completion of the passage and (ii) the rated plausibility of the passage as a whole. The authors conclude that this analysis suggests that for unpredicted lexical items both coherence (plausibility) with the preceding discourse and activation of overlapping semantic features reduced the amplitude of the N400 and that the time difference suggests that there is no single point during lexical processing when all potential constraints affecting word processing simultaneously come to bear.

Common to all studies discussed above is that they looked at the effects of plausibility (or semantic similarity) on unpredictable, “low-cloze” words. As noted by Nieuwland et al. (2019, p. 5), these studies, therefore, do not directly address the question of whether or to what extent the well-established, graded relationship between predictability and N400 activity is confounded by other contextual semantic factors. For example, possible correlations between predictability, plausibility, and semantic similarity can make it difficult to establish their effects on semantic processing. To overcome this weakness, Nieuwland et al. (2019) examined the effects of predictability, plausibility, and semantic similarity across a full range of cloze values.^7^ They simultaneously modeled variance associated with the three measures allowing them to investigate the effects of one variable (measure) while controlling for the others. Predictability was determined using a cloze test, and plausibility was computed using a norming test based on a 7-point scale. On average, high predictable nouns were rated as plausible whereas low predictable nouns were rated as neither plausible nor implausible. The authors found that effects of predictability and plausibility both occurred in the N400 time window, but the former dominated the rise of N400 (i.e., upward flank), while the latter set in at its fall (i.e., its downward flank). By contrast, semantic similarity [calculated using both LSA and Snout, a word2vec-compatible ‘continuous bag of words’ (CBOW) prediction-model] did not have a strong effect on N400 activity over and above the effects of predictability and plausibility. Importantly, they found that even when accounting for the possibility that plausibility and semantic similarity have stronger effects for relatively unexpected words, plausibility modulated activity of the N400 after the peak effect of predictability (Nieuwland et al., 2019, p.18).

### 1.3. Plausibility of Event Structures or Typicality Between Categories?

If N400 activity is not only characterized by predictability but also by plausibility, the question arises how the plausibility component can be defined. In order to answer this question, the following two questions have to be answered: (i) do pre-activated features play a role, and (ii) what concepts are involved? Pre-activated features are related to an (undischarged) argument of the current event structure. The corresponding concept is *C*_*arg*_. The event structure is related to the concept *C*_*e*_ (which is of type “event”). Finally, *C*_*CW*_ is the concept expressed by CW. If pre-activated features play no role, this means that *C*_*arg*_ is not involved in the definition of the plausibility component. Plausibility is defined as the plausibility of the update of *C*_*e*_ with *C*_*arg*_. This way of defining the plausibility component will be called the Strict Plausibility Hypothesis. If pre-activated features play a role, *C*_*arg*_ is involved. Two possibilities must be distinguished. According to the first possibility, plausibility is computed in two steps. *C*_*e*_ is first updated by *C*_*arg*_ to $C_{e}^{\prime }$ and than the plausibility of $C_{e}^{\prime }$ with *C*_*CW*_ is computed. Updating *C*_*e*_ with *C*_*arg*_ possibly changes the probabilities of which nouns are expected and hence which nouns yield a (most) plausible event structure. This will be called the Plausibility-cum-Prediction Hypothesis. Common to this hypothesis and the first one is the assumption that it is the plausibility of an event structure that is computed. This is in contrast to the third hypothesis that corresponds to the second possibility. Plausibility is defined in terms of an operation on *C*_*arg*_ and *C*_*CW*_. On this account, pre-activated features act directly through semantic memory without an intermediate step relating them to *C*_*e*_ (for a similar view see Paczynski and Kuperberg, 2011). As a result, plausibility of an event structure plays no role.

One way of testing the three hypotheses is to introduce a feature or a set of features of the CW before this word is encountered. Importantly, this feature (or set of features) is not in accordance with features that have already been pre-activated so that a mismatch between the newly and the previously activated features results. In the study two different strategies have been used to test these hypotheses. The first strategy uses an induced prediction. Before the target sentence, the comprehender is told that a particular word will occur in the continuation, and unbeknown to her, this word is the CW. The second strategy uses prenominal elements in an NP of which the CW is the head noun. Examples of prenominal elements are adjectives and determiners.

The first strategy was used by Szewczyk and Schriefers (2018). They used two types of scenarios. The context for both scenarios was the same. In the first type, this context was followed by the target sentence in which the CW was either plausible or implausible given the preceding context. In the second type, the target sentence was preceded by a sentence in which an explicit prediction was introduced. A comprehender was told that the particular word X would be used in the following text. This word was identical with the CW. Hence, there were four conditions by crossing induced vs. non-induced prediction with the factor “(im-)plausible.” An example is given below in (11).

**Table:** 

(11)	a.	Context: My uncle loves to make practical jokes. During the last summer he mounted a triangle fin on his back, jumped into the water and approached the swimming area with his fin only above the water.
	b.	Induction of prediction: In the upcoming sentence you will see the following word: “shark” / “doctor.”
	c.	Target sentence: There was terrible fuss and everybody thought they saw a shark / doctor approaching them.

The authors found an N400 only in the no-induced-non-plausible condition. In the other three conditions, no N400 was observed. These results are incompatible with the Strong Plausibility Hypothesis. According to this thesis, there should be a difference in N400 amplitude in the two induced prediction conditions. The prediction component yields the same results because *C*_*arg*_ = *C*_*CW*_ in both conditions. Since the induced prediction does have no effect on the plausibility of the resulting event structure, the CW “shark” results in an event structure that is more plausible than the event structure that results if “doctor” is encountered. However, the N400 amplitudes did not differ in the two conditions. The results are compatible with the other two hypotheses. Let us start with the Plausibility-cum-Prediction Hypothesis. Processing the explicit prediction in the incongruent condition changes the expectations with respect to which event structure is described. Prior to the prediction, an event structure was expected in which sharks participate, e.g., that a shark is approaching the swimming area. This expectation is changed by the induced prediction “doctor,” which has the effect of raising the probability of an event structure in which a doctor participates (and lowers the probability of an event structure in which a shark occurs). Compatibility with the Typicality Hypothesis is shown as follows. According to this thesis, the plausibility component is modeled as an operation on *C*_*arg*_ and *C*_*CW*_. Since one has *C*_*arg*_ = *C*_*CW*_, no N400 is expected.

Let us next turn to studies that allow for distinguishing between the two other hypotheses. Recall from section 1.1 that Boudewyn et al. (2015) found for examples like those in (12) the following ranking of N400 amplitudes: “sweet and tasty cake” < “healthy and tasty cake” < “healthy and tasty veggies” < “sweet and tasty veggies.”

**Table:** 

(12)	Frank was throwing a birthday party, and he had made the dessert from scratch. After everyone sang, he sliced up some sweet/healthy and tasty cake/veggies that looked delicious.

The results are incompatible with the Plausibility-cum-Prediction Hypothesis. Encountering “healthy,” changes the expectations of the kind of birthday party that is being described. Now a comprehender expects a birthday party that is atypical at least with respect to some food that is served. Healthy food becomes the most expected food in this context. As a result, “healthy and tasty veggies” should elicit an N400 amplitude that is not smaller than that for “healthy and tasty cake.” By contrast, the results are compatible with the Typicality Hypothesis. The context pre-activates features of food that is typically served at a birthday party. Encountering “healthy,” *C*_*arg*_ is updated because the corresponding feature is added to this concept. Cake is still an expected food. However, it is now not the most typical kind of this sort because being healthy is an atypical property of cakes.

According to the Typicality Hypothesis, a “mismatching” prenominal element targets only *C*_*arg*_. Before the prenominal element is encountered, a particular set of objects falling under this concept is expected most. The effect of a mismatching prenominal element is to change this expectation to a different set. As a result, nouns that were unpredictable before become (more) predictable afterward. According to this thesis, the effect of a mismatching element is, therefore, purely prediction-driven and not related to the plausibility of event structures. By contrast, according to the Plausibility-cum-Prediction Hypothesis, not only *C*_*arg*_ is changed but also *C*_*e*_. This latter change is related to the plausibility of the event structure. Hence, it is, at least in part, plausibility-driven. This raises the question of whether there is neural evidence that allows for distinguishing between the two hypotheses. According to the Plausibility-cum-Prediction Hypothesis, a mismatching pre-nominal element should trigger a revision that is driven by the overall plausibility of the continuing text and, therefore, of the resulting event structure. By contrast, according to the Typicality Hypothesis, the revision should be driven by a revision that only targets *C*_*arg*_ and, hence, the predictability of an upcoming noun, independently of the plausibility of the resulting event structure. This question was investigated in Fleur et al. (2020). The authors investigated pre-nominal effects in Dutch definite NPs. In Dutch, definite articles (“de / het”) are marked for gender. One hypothesis tested by the authors was the “noun prediction revision hypothesis.” According to this hypothesis, comprehenders predict the noun (with or without its gender) and then use article gender, once available, to revise the noun prediction. They used scenarios that strongly predicted a definite NP as its best continuation, followed by a definite NP with the expected noun or an unexpected, different gender NP. An example is given in (13).

**Table:** 

(13)	Het is zondagochtend. De gehele gelovige familie gaat zoals altijd naar de kerk / het gebedshuis in het dorp.It is Sunday morning. The whole religious family goes, as always, to the church / the worship place in the village.

The authors found that gender-mismatching articles elicited increased N400 activity compared to matching articles, consistent with several other studies (see Fleur et al., 2020 for references). A second question that was addressed by the authors was whether mismatching articles caused comprehenders to revise their noun prediction instead of simply dropping it. Such a revision process could be correlated with the contextual constraint toward one alternative continuation. For example, encountering “het” in (13) instead of “det” a comprehender may revise his prediction to “gebedshuis.” This revision should show up in two effects. First, there should be an effect in the neural response to gender-mismatching articles, and second, a successful revision should facilitate the processing of the corresponding noun that should be reflected in an attenuated N400 amplitude. Prediction revision at the article was quantized as next-word entropy on article-elicited ERPs in the 500–700 ms time window. Revised predictability of nouns was quantized as cloze probability of the prediction mismatching nouns given a gender-mismatching article. The authors found that next-word entropy on article-elicited ERPs correlated with revised predictability, i.e., more predictable nouns elicited smaller N400 amplitudes. Importantly, since other factors like semantic similarity to the (originally) predicted noun and plausibility of the resulting sentence were controlled for, the reduction in the N400 amplitude can be attributed to a revision of a prediction and not to semantic similarity to the initially predicted noun or the overall plausibility of the sentence.^8^ When taken together, the studies Boudewyn et al. (2015) and Fleur et al. (2020) provide evidence for the Typicality Hypothesis and against the Plausibility-cum-Prediction Hypothesis.

## 2. A Theoretical Account of the Hybrid View for the N400

The preceding sections have provided evidence that (i) N400 activity is functionally characterized by two different components: predictability and plausibility; (ii) both components are operations on *C*_*arg*_ and *C*_*CW*_; (iii) the predictability component cannot be defined in terms of feature overlap between *C*_*arg*_ and *C*_*CW*_; and (iv) from (ii) it follows that the plausibility component is not related to the plausibility of an event structure.

In order to give a theoretical model of a hybrid account, both the predictability and the plausibility components have to be defined. For the predictability component, the central question is as follows: how exactly is retrieving features from long-term memory linked to the modulation of the N400 amplitude? Since retrieving information is related to prediction, the link to N400 activity should be defined in terms of a function of pre-activated and actually found features. For the plausibility component, the corresponding question is as follows: what is the target into which pre-activated and non-pre-activated features get integrated and how is this operation defined? An answer to this question must take into account that the N400 is only *one* ERP component that is linked to semantic processing. More specifically, one has to distinguish the integration operation related to N400 activity from that (or those) related to brain activity in the post-N400 time window, in particular to late positivities.

### 2.1. Plausibility and Typicality

One, if not the most important cognitive role of categorization, is to allow for generating (default) inferences. As Holland et al. (1986) put it: “To know that an instance is a member of a natural category is to have an entry point into an elaborate default hierarchy that provides a wealth of expectations about the instance.” This can be illustrated with an (in-)famous example from Artificial Intelligence (AI). If someone learns that Tweety is a bird, then using her knowledge that birds normally fly, she will (defeasibly) infer that Tweety can fly. Such default inferences not only apply to categories expressed by common nouns like “bird” but also to the categories associated with argument positions in event structures and scenarios. More specifically, one has that each critical word expresses a category. Similarly, each argument position of a verb is associated with a (most specific) category and in each scenario each event or state denoting expression is associated with a (most specific) category. For example, in scenario (1) two default inferences for the theme of the planting event is that its habitat are the tropics and that it is tall. Default inferences are, at least in general, context-dependent and, hence, non-monotonic. If a comprehender later comes to know that Tweety is in effect a penguin, the default inference that he can fly will be given up. Similarly, if she learns that, in effect, pines and not palms were planted, she has to withdraw the inference that the habitat are the tropics. What triggers such inferences is the graded structure of categories. Features that belong to a category are not equivalent with respect to category membership in the sense that they represent necessary and sufficient conditions for objects to belong to the category but are assigned weights that reflect their discriminative value for the category. Hence, objects falling under a category vary in how good an example or how typical they are of the category (see Barsalou, 1985 for discussion and references). For example, the ability to fly is a typical property of birds and the property of being found in the tropics is a typical property of objects that do or should look tropical. A direct consequence of this difference in typicality is that features in the representation of the CW differ in the way they fit into the feature structure given by the pre-activated features. Even if they match with one of those features, the typicality of the feature must be taken into account.

This graded structure is not invariant but is highly dependent on constraints inherent in specific situations and contexts, (Barsalou, 1987, p. 107). As an effect, not all features of objects are relevant in a particular scenario but only a particular subset. One reason for this partial character of categories in contexts is that objects are usually used to achieve particular goals or are involved in prerequisites or consequences of actions that are undertaken to achieve such goals. For example in (14) taken from Chwilla et al. (2007), the paddles or Frisbees are used to dislocate water in order to move a canoe in the water. Hence, the important similarity between Frisbees and paddles is that they are typically made of a solid material. By contrast, the fact that pullovers share with paddles the property of being prototypically made of some biological material (wool and wood) plays no role. This relation between a goal and the relevant properties for achieving it is reflected in the N400 amplitude. It is larger for “pullovers” than for “Frisbees.”

**Table:** 

(14)	The boys found a canoe in the spare room. With this, they wanted to go canoeing on the canal whatever the costs. The fact that they could not find the paddles did not lead them to make up their mind. According to the boys, you do not at all need them. They let the canoe into the water and paddled with Frisbees / pullovers.

In the scenario (1), the objects planted along the driveway are chosen in such a way that they have the effect of making the resort look tropical because this was the ultimate intention of the owners. Consider as a further illustration the following example from Roth and Shoben (1983). The authors let participants read pairs of sentences like those in (15).

**Table:** 

(15)	a.	1st sentence: Stacy volunteered to milk the animal whenever she visited the farm.
	b.	1st sentence: Fran pleaded with her father to let her ride the animal.
	c.	2nd sentence: She was very fond of the cow / horse.

In order to understand the second sentence of the scenario in (15-c), a comprehender must establish an anaphoric link between “cow” or “horse” and “animal” in the first sentence. Both expressions are co-referential, i.e., they refer to the same object (animal). Using reading times on the CW, the authors found that in the context of (15-a) “cow” was facilitated compared to “horse.” By contrast, in the context of (15-b) the facilitation effect was reversed. One way of explaining these findings is to assume that “animal” had a different graded structure in the two examples. Whereas, cows and goats are typical examples of animals in the first context, horses and mules are typical examples in the second one. This is the case because different properties of the category “animal” are activated on two occasions. In the case of (15-a), features like milkable and lives_on_farm are activated, whereas in the context of (15-b) rideable is activated. One way of modeling this context dependency of categories was suggested in Barsalou (1983). On a given occasion of use, only a subset of the properties associated with a category is usually activated. This active subset contains the following: (i) context-independent properties that are active on all occasions the concept is processed, and (ii) context-dependent properties that are activated only in relevant contexts. Such context-dependent uses of concepts will be called category concepts. In this study, we use “context-independent” as synonymous with “selection restrictions” imposed by a verb. For example, “caution” imposes on its theme both animacy constraints and the constraint that this object be in (some kind of) danger.

An example from the ERP study that was already discussed above further illustrates this distinction. For the verb “caution,” animacy features are context-independent both for the actor and the theme argument. In addition, the theme argument satisfies the further selection restriction “in_danger,” which too is context-independent because it is activated in every context in which this verb is used. Depending on the context in which the verb is used, additional context-dependent features can be imposed. For example, in the context of a seaside scenario like that in (2-a) features like in_water and afloat are added to those pertaining to animacy and other selection restrictions like “in_danger” to the theme argument. By contrast, in the LC scenario (2-b) these context-dependent features are not added.^9^

Let us relate the above considerations to N400 activity. *C*_*arg*_ is a category concept. The (pre-activated) features of *C*_*arg*_ are default inferences that are licensed either by the category underlying *C*_*arg*_ (context-independent) or by the context in which *arg* occurs (context-dependent features). *C*_*arg*_ extends the information about the current scenario and the current event, more specifically, adding *C*_*arg*_ to *C*_*event*_ leads to an extension of *C*_*event*_, say $C_{event}^{\prime }$, in which *C*_*arg*_ is embedded. *C*_*CW*_ is not a category concept because so far it has not yet been situated in the sense that it has been combined with the current context. This is carried out by the update operation that combines *C*_*arg*_ with *C*_*CW*_. During this update process, the typicality of the features in *C*_*CW*_ that corresponds to features in *C*_*arg*_ is computed. The more typical these features are to those in *C*_*arg*_, the more attenuated is the N400 amplitude. Hence, on this definition of the plausibility component, plausibility is, in effect, typicality. The computation of typicality can be seen as locating *C*_*CW*_ in the graded structure of *C*_*arg*_. One way of viewing this “localizing” is to take it as an operation that (partially) “integrates” *C*_*CW*_ into *C*_*arg*_. The refined thesis about the plausibility component is given in (16).

**Table:** 

(16)	The plausibility component of N400 activity is related to a typicality computation: how typical are the features in *C*_*CW*_ that correspond to a feature in *C*_*arg*_ to those in *C*_*arg*_?

### 2.2. Predictions and Information Gain

Given a context *c* consisting of the words *w*_1_…*w*_*n*_ a set of pre-activated features belonging to *C*_*arg*_ related to *w*_*arg*_ ∉ *c* is given. Before the CW is encountered, the information in *C*_*arg*_ is not confirmed by bottom-up information. If CW or a prenominal element related to CW is encountered, the information in *C*_*arg*_ is so to speak tested against the empirical bedrock in form of bottom-up information. The result of this testing operation is the information gain (or prediction error) relative to *C*_*arg*_.

The question arises of how this test operation can be defined. If categories are based on a bi-valent taxonomic hierarchy, the answer is simple. Given a feature *f* in *C*_*arg*_, it is confirmed (success of prediction) if it is also in *C*_*CW*_ and it is disconfirmed (prediction error) if it is not in *C*_*CW*_. However, this model does not take into account the situated and partial character of predictions. What gets predicted is only a small subset of the set of features that are appropriate for objects falling under a category. Hence, for most of the features neither *f* nor its negation is an element of *C*_*arg*_. Let us make this precise. For a given category concept and a feature *f*, three cases must be distinguished: *f* is an element of the category concept, its negation is an element of the category concept, or neither *f* nor its negation is an element of this category concept. If *f* (the negation of *f*) is an element both of *C*_*arg*_ and *C*_*CW*_, *f* is said to be confirmed by *C*_*CW*_. If *f* is an element of *C*_*arg*_ whereas its negation is an element of *C*_*CW*_, *f* is said to be disconfirmed by *C*_*CW*_. Similarly, if the negation of *f* is in *C*_*arg*_ but *f* is in *C*_*CW*_, *f* is said to be disconfirmed by *C*_*CW*_.

The interesting case arises if there is a default inference in *C*_*arg*_ but no corresponding inference in *C*_*CW*_. If there is a default inference in *C*_*arg*_, this means that in the particular context that gives rise to this category concept it is likely that the object has this property. Consider, e.g., (2-a). In this scenario upon processing the verb “caution,” there is a default inference for the attribute in_danger and the value “swimmers” because in this particular context it is highly likely that the swimmers will be cautioned by the lifeguards. To put it differently, *C*_*arg*_ can be taken as a (situated) category concept for swimmers that extends (or situates) *C*_*swimmers*_. In this case too, the feature *f* is said to be confirmed by *C*_*CW*_. What happens for sorts like “trainees” for which there is no corresponding default inference in *C*_*arg*_ for the attribute in_danger, i.e., for which neither *f* nor its negation is an element of the category concept? Given the context, it is not likely that trainees are in danger. However, there is an extension of *C*_*arg*_, say $C_{\text{arg}}^{*}$, in which the inference is licensed, which can be taken as a category concept of *C*_*CW*_. For example, if scenario (2-a) is continued by the information that the sharks were seen in a location close to that in which trainees were bathing, this probability will be high for this sort of object. Hence, one not only considers the current *C*_*arg*_ but also possible extension of it. If there is an extension that licenses the inference for *C*_*CW*_ because this extension is a (situated) category concept of *C*_*CW*_, the default inference in *C*_*arg*_ will be said to be compatible with *C*_*CW*_.

Hence, we arrived at a three fold distinction: confirmed, disconfirmed, and compatible. The information gain relative to *C*_*arg*_ can be defined in two different ways. One can take only those features that are confirmed by *C*_*CW*_. This excludes both mismatched and (only) compatible features. Alternatively, this gain can include in addition to the confirmed features also the compatible ones. We suggest that for N400 activity the latter definition is correct. There are at least two reasons for this. First, as shown above, compatible features can be confirmed at a later stage of the discourse and given the fact that the speaker introduced them into the discourse, it is likely, provided she is reliable (rational). The second argument is related to a peculiarity of the N400. It is not a direct index of prediction violation. Its amplitude for “trainees” is the same in the HC scenario (2-a) and in the LC scenario (2-b). As will be shown below in section 3, in order to account for this sameness, compatible features need to be part of the information gain. Our hypothesis about the predictability component of N400 activity is given in (17).

**Table:** 

(17)	The predictability component of N400 activity is related to the information gain relative to *C*_*arg*_ defined as the set of pre-activated features in this category concept that are not disconfirmed by *C*_*CW*_.

Whereas predictability focuses on *C*_*arg*_: which features in this category concept are not disconfirmed?, plausibility focuses on *C*_*CW*_: how typical are the features in this category in relation to *C*_*arg*_? Hence, on our view of a hybrid approach to N400 activity, the whole process comprises three steps, two of which characterize N400 activity. In the first step, the context determines a category concept *C*_*arg*_ to which belong both context-independent features determined by the underlying category and context-dependent features that provide information about the category in this particular context. If the CW is encountered, *C*_*arg*_ and *C*_*CW*_ must be combined with each other. This update operation comprises two steps that are related to N400 activity. First, the information gain in terms of non-disconfirmed features of *C*_*arg*_ is computed (predictability component) and next the typicality of features in *C*_*CW*_ that have corresponding features in *C*_*arg*_ is computed (plausibility component).

## 3. Outline of a Formalization

Pre-activated features in *C*_*arg*_ represent default inferences that are either licensed by the underlying category (context-independent) or by the embedding context (context-dependent). Let this set be Ω. Encountering *C*_*CW*_ triggers an update operation that combines the two concepts, yielding a combined category concept. This resulting category concept is computed in two steps. In the first step, the set Ω is split into three disjoint sets: the set of confirmed features Σ_*conf*_, the set of compatible features Σ_*comp*_, and the set of disconfirmed features Σ_*disconf*_. In the second step, the resulting category concept is construed using the result of the first step. The first step is related to the predictability component, and the second step to the plausibility component. N400 activity is functionally characterized by the properties of the two operations. For the first step, this is the entropy reduction triggered by Σ_*conf*_ and Σ_*comp*_, and for the second step, this is the typicality of *C*_*CW*_ relative to *C*_*arg*_. In this section, we will sketch how these ideas can be made formally precise.^10^

### 3.1. Concepts as Frames

The first task is to find an appropriate representational format for categories. From what has been said so far it follows that there are three principle constraints that such a format must account for: (i) the internal structure in terms of features; (ii) the graded structure in order to allow for the definition of similarity (of values) and salience (of attributes); and (iii) the context-dependent use of categories. An appropriate representational format that allows for the satisfaction of these constraints is frames. Frames are built out of attribute-value pairs. Such pairs have been called features or properties in the sections above. The value space of an attribute is sorted, i.e., an attribute can take values only in a particular set which is the sort of the attribute. The structure of frames is recursive, i.e., the value of a frame can be a frame so that this value can be specified by further attributes. Each frame is of a particular sort. Sorts are not restricted to those associated with common nouns like “fruit,” “apple,” or “dog” but also include sorts associated with verbs and their arguments (e.g., theme or actor) as well as sorts for scenarios (or scripts) like “seaside” or “going to a restaurant.” The relation between a frame and the chains of attributes belonging to it is captured by a function θ. One has θ(*f*) = Σ if Σ is the set of features, i.e., the set of chains of attributes together with their values belonging to *f*. In this study, we will denote a feature consisting of an attribute (chain) *A* with value *V* as *VA*. On frames of a particular sort σ, an information ordering ⊑_σ_ is defined. One has $f{⊑}_{\sigma }f^{\prime }$ if each chain of attributes that is defined for *f* is also defined for *f*′, and the value of the chain in *f* subsumes the value of the chain in *f*′. The information ordering and the frame hierarchy it induces can be used to account for the context-dependent use of categories. A category of a particular sort can be represented by the whole hierarchy of that sort. The use of a category in a particular context, i.e., a category concept, is represented by an element in this hierarchy so that only a particular subset of the (chains of) attributes is activated, (for a more detailed presentation of the underlying frame theory, see Naumann and Petersen, 2019). Frames in which a (chain of) attributes is assigned its value space together with a probability distribution on this space are stochastic frames (cf. Naumann et al., 2018).

### 3.2. Weights on Values, Probabilities, and Default Inferences

One strategy of defining weights on values is to assume that in category concepts attributes are not assigned a particular value but a data structure containing values that are weighted by typicality (see Cohen and Murphy, 1984 and the approach by Smith et al., 1988 for a similar proposal). One way of making this idea of a data structure formally precise in a frame theory has been suggested in Schuster (2016) (see also Schurz, 2012). Instead of assigning an attribute a particular value, it is assigned its value space together with a probability distribution on this space. In particular, each value *V* in the value space of an attribute *A* that belongs to a category *C* is assigned a (conditional) probability *P*(*VA*|*C*), i.e., the probability of *VA* given *C*. These conditional probabilities can be taken to reflect subjective conditional probabilities of a comprehender that are based on his world knowledge and linguistic knowledge based on statistical regularities in texts and discourses.

Having weights on values, one can define which features belong to a category or a category concept. Recall that default inferences belong to a category concept. What is required, therefore, is a link between probability distributions and default inferences. One way to relate default inferences to probabilities was suggested by Schurz (2012). A default inference or normic conditional of the form “Cs normally have P” or “Cs are normally Ps” (formally *C* ⇒ *P*), e.g., “Birds (can) normally fly” or “Cake is normally sweet and unhealthy” only holds if the corresponding conditional probability is high. This is summarized in the statistical consequence hypothesis (SC), (Schurz, 2012, p. 531).

**Table:** 

(18)	SC:	*C* ⇒ *P* implies that the conditional statistical probability of *P* given *C*, *P*(*P*|*C*) is high.

In our framework, one has *C* ⇒ *VA* if *P*(*VA*|*C*): = *max*(*P*(*V*_1_*A*|*C*), …, *P*(*V*_*n*_*A*|*C*)) and *P*(*VA*|*C*) > *r*. Thus, a normic conditional holds for a feature *VA* in a category *C* if its conditional probability is the maximum of the conditional probabilities of the *n* values of the value space. The constraint that the probability is high is defined by the requirement that *P*(*VA*|*C*) > *r* for some threshold value *r*, e.g., *r* > 0.5.^11^

How does the SC hypothesis relate to categories and category concepts? Recall that to a category concept belong both context-independent and context-dependent features. For example, for the category concept associated with the theme of “caution” context-independent features are be_in_danger and features related to animacy. These features are determined by the underlying category because they do not depend on the context. For this reason, they always belong to a category concept, (see Barsalou, 1983 for discussion). Context-dependent features result from correlations in the following way. In a category of a scenario or an event, the values of attributes are, in general, not independent of each other. Rather, there are correlations between these features [or the values of (chains of) attributes]. For example, in the holiday resort scenario in (1) the information that the resort should look tropical and that something was planted along the driveway triggers the default inference that the habitat of the objects planted is most likely the tropics and that they are tall in order to be visible. Hence, the inference has the form *C*_*script*_ ⇒ *VA* or *C*_*event*_ ⇒ *VA*. In our application, *VA* is always a feature in the category concept *C*_*arg*_ associated with an argument of the scenario or the event that has not yet been discharged. *C*_*script*_ or *C*_*event*_ provides the context in which the category concept *C*_*arg*_ is processed.

### 3.3. The Predictability Component and Entropy Reduction

Recall that we hypothesize that the predictability component is related to the information gain of pre-activated features in *C*_*arg*_ that are not disconfirmed by *C*_*CW*_. Since the first step is input to the second step, this first step is defined in such a way that it yields three sets of features: Σ_*conf*_, Σ_*comp*_ and Σ_*disconf*_. We formalize this first step as the operation update_set, which takes two categories and returns a triple of sets of features. update_set(*C*_*arg*_, *C*_*CW*_) is a partial function; it is defined only if the chain of attributes for every feature in *C*_*arg*_ is also defined for *C*_*CW*_. If this function is defined, the update operation is defined as follows: update_set(*C*_*arg*_, *C*_*CW*_) = 〈Σ_*conf*_, Σ_*comp*_, Σ_*disconf*_〉 iff for each *VA* ∈ *C*_*arg*_: if *VA* ∈ *C*_*CW*_, then *VA* ∈ Σ_*conf*_; if $V\neq \bar{V}$ and ${\bar{V}}^{A}\in C_{\text{CW}}$, then $V^{A}\in \Sigma_{\text{disconf}}$; if $V^{A}\notin C_{\text{CW}}$ and $V^{A}\notin \Sigma_{\text{disconf}}$, then $V^{A}\in \Sigma_{\text{comp}}$. One has: *VA* ∈ *C*_*arg*_ ∧ *VA* ∈ *C*_*CW*_ iff *P*(*VA*|*C*_*arg*_) > *r* and *P*(*VA*|*C*_*CW*_) > *r*; an example is habitat = tropics in the holiday resort in (1) for the CW “palms.” $VA\in C_{\text{arg}}\wedge \bar{V}A\in C_{\text{CW}}$ iff *P*(*VA*|*C*_*arg*_) > *r* and $P\left(\bar{V}A|C_{\text{CW}}\right)>r$ for two different values *V* and $\bar{V}$. An example is habitat = tropics in *C*_*arg*_ and habitat = moderate in *C*_pine_. An example where a feature is in *C*_*arg*_ but not in *C*_*CW*_ is location = water in the seaside scenario in (2-a) for the CW “trainees.”

The computation of the three sets fails if an attribute in *C*_*arg*_ is encountered that is not defined for *C*_*CW*_. An example is “drawer” in scenario (2-a), as for its associated category, animacy attributes are not defined. In our approach, this failure has the effect that typicality is not computed. We will come back to this point below. Though Σ_*conf*_, Σ_*comp*_, and Σ_*disconf*_ are sets, they are uniquely related to frames (categories). For example, one has θ(*f*_Σ_*conf*__) = Σ_*conf*_, i.e., *f*_Σ_*conf*__ is the (unique) frame to which the features in Σ_*conf*_ belong.

An alternative view on Σ_*conf*_ ∪ Σ_*comp*_ is as a measure of prediction error. The smaller this set is, the higher is the prediction error, or, using the gain in information: the smaller the gain in information in non-disconfirmed features, the higher is the prediction error. By itself, Σ_*conf*_ ∪ Σ_*comp*_ does not measure the information gain of non-disconfirmed features in *C*_*arg*_. We suggest that this gain can be measured by entropy reduction, which is related to the information-theoretic measure of entropy. More generally, two theoretical metrics that have been used to measure information are surprisal and entropy. Surprisal quantifies how unexpected a state *s* is given a context *c*. Entropy quantifies how uncertain a system is about what comes next so that it derives from the probabilities of all future states (Willems et al., 2016). From these characterizations, it follows that surprisal is backward-looking: given a context *c*, how likely is it to encounter a state *s* or how likely is the updated context *c*^⊓^*s*? By contrast, entropy is forward-looking. It quantifies the reduction in uncertainty about the current state the system is in. This difference is also reflected in the definition of the two metrics. Given a state *s*_*t*_ with predecessors *s*_1_…*s*_*t*−1_, surprisal at *t* is defined as the negative logarithm of the conditional probability of *s*_*t*_ given *s*_1_…*s*_*t*−1_.

**Table:** 

(19)	surprisal(*t*): = −log*P*(*s*_*t*_|*s*_1_…*s*_*t*−1_).

By contrast, entropy is not a function of the probability of the state at *t* but of the distribution of probability of all future states.

**Table:** 

(20)	$H\left(t\right):=-\underset{s_{t+1}\in S}{\sum }P\left(s_{t+1}|s_{1}\ldots s_{t}\right)\log P\left(s_{t+1}|s_{1}\ldots s_{t}\right)$.

Given this forward-looking character of entropy, one often is interested in entropy reduction. Given two states *s*_*t*_1__ and *s*_*t*_2__ at *t*_1_ and *t*_2_, respectively, entropy reduction triggered by state *s*_*t*_2__ is defined as the difference in entropy between *t*_1_ and *t*_2_.

**Table:** 

(21)	Δ*H*(*s*_*t*_2__): = *H*(*t*_1_) − *H*(*t*_2_).

The higher entropy reduction is, the more information is gained relative to non-disconfirmed features. The relation to prediction error is the following. The lower entropy reduction is, the higher is the prediction error.

We hypothesize that there is the following relation between the two measures and the two processing components. Entropy reduction is related to the predictability component, whereas surprisal is related to the plausibility component. According to our account, the predictability component is related to the gain in the information of non-disconfirmed pre-activated features. This information gain can be taken to be given by the reduction in uncertainty about the category that is expressed by the CW. By contrast, the computation of typicality, i.e., the location of *C*_*CW*_ in the graded structure of *C*_*arg*_, can be taken as reflecting how (un)expected the features in *C*_*CW*_ are given *C*_*arg*_, which comprises the influence of the context on *C*_*CW*_.

In our approach, entropy is defined on the frame hierarchy of frames of a particular sort, e.g., “swimmer” or the theme of caution events. Due to the recursive character of frames, there is, at least in principle, no upper bound on the length of chains in a frame, though for a particular frame there always exists such a bound. Hence, considering arbitrary extensions would make the computation of entropy reduction intractable. We suggest to consider *n*-step extensions, i.e., frames in which the maximal length of chains is *n*. The minimal case are frames in which all chains have length 0. These are minimal frames in the sense that only information about the sort of the frame is supplied but no relational information that links the referent of the frame to other objects. In our application, *n* will, in general, be low, say *n* = 2 or *n* = 3. Let us next define entropy in our approach. For a given frame hierarchy ⊑_σ_ of sort σ, let $F_{{⊑}_{\sigma }}^{n}$ be the set of frames in which the maximal length of chains is *n* and let *f*_*t*_ be the frame at *t*. Entropy at *t* is then defined as given in (22).

**Table:** 

(22)	$H\left(t\right):=-\underset{f^{n}\in F_{{⊑}_{\sigma }}^{n}}{\sum }P\left(f^{n}|f_{t}\right)\log P\left(f^{n}|f_{t}\right)$.

According to this definition, entropy is 0 if *f*_*t*_ singles out a unique frame of length *n*, i.e., a unique category concept of this length. This is the case if *f*_*t*_ specifies values for all chains of length less or equal *n*. This will most likely never be the case for the simple reason that it goes against the context dependence of category concepts. For entropy reduction, one considers *f*_Σ_*conf*_ ∪ Σ_*comp*__ at *t*_2_, i.e., one has *f*_*t*_2__ = *f*_Σ_*conf*_ ∪ Σ_*comp*__, i.e., the frame (category) that corresponds to the set of confirmed and compatible features. What is the frame (category) at *t*_1_? This is the frame containing the features already got for the argument position before the CW is encountered. An example is the scenario of the birthday party involving sweet or healthy cake in (8) where one or more features of the category concept associated with the CW “cake” are determined by preceding adjectives. If no bottom-up information is given, one possibility is to consider a minimal frame of the given category that only contains sortal information. However, this does not account for the fact that there are possible discourse-independent default inferences in the category like those related to animacy in the category of the theme of caution events. We, therefore, suggest that at *t*_1_ one uses a minimal frame that is closed under such context-independent default inferences.

### 3.4. The Plausibility Component and Typicality

The second step is executed only if the operation associated with the predictability component did not yield failure. If the first step was successful, the second step consists in building up the final category concept. This is formalized by an operation update_*t*_(Σ_*conf*_, Σ_*comp*_, Σ_*disconf*_), which takes three sets of features and returns a frame (category). This operation is defined as follows: update_*t*_(Σ_*conf*_, Σ_*comp*_, Σ_*disconf*_) = *f*_Σ_*conf*_ ∪ Σ_*disconf*__. Note that features in Σ_*conf*_ are features that are default inferences in both *C*_*arg*_ and *C*_*CW*_ and are thus taken over to update_*t*_(Σ_*conf*_, Σ_*comp*_, Σ_*disconf*_) because the feature in *C*_*arg*_ is confirmed by the corresponding feature in *C*_*CW*_. However, features in Σ_*comp*_ that are only compatible, i.e., which are in *C*_*arg*_ but not in *C*_*CW*_ are not taken over to update_*t*_(Σ_*conf*_, Σ_*comp*_, Σ_*disconf*_) because, at least at this stage, they are not confirmed by the bottom-up information given by *C*_*CW*_. An example is be_in_danger in the scenario (2-a) for “trainees.” If instead of “trainees” “swimmers” is encountered, the situation is different. In this case the default inference is licensed for this sort of objects so that it is taken over to the resulting category concept. Elements of Σ_*disconf*_ are taken over. They are context-independent default inferences in *C*_*CW*_ that disconfirm the corresponding feature in *C*_*arg*_. An example is the features habitat = moderate and height = small in scenario (1) if the CW is “tulip.”

The property of this operation by which the plausibility component of N400 activity is functionally characterized is typicality of *C*_*CW*_ relative to *C*_*arg*_. Typicality is defined in terms of weights on values (similarity) and weights on attributes (diagnosticity).

### 3.5. The Definition of Similarity and Diagnosticity

Similarity between features of the category concept and the representation of the CW is defined in terms of the weights on values, i.e., the conditional probabilities. We follow Schuster (2016) whose definition is inspired by that of Smith et al. (1988) and define this similarity for a feature as the minimum probability of this feature for *C*_*arg*_ and for the corresponding value in the representation *C*_*CW*_ of the CW.

**Table:** 

(23)	*sim*(*C*_*CW*_, *C*_*arg*_|*VA*) = *min*(*P*(*VA*|*C*_*arg*_), *P*(*VA*|*C*_*CW*_)).

Using the minimum of the value in the category concept and the value in the representation of the CW ensures that probabilities are considered at least as strongly as the values of the category concept and at least as much as the value of *CW*. Next, we turn to the definition of diagnosticity.

Statistical frequency does not account for the fact that features with the same (high) frequency can differ in the way they can contribute to the categorization process. What is required, therefore, is a measure that specifies the discriminative value of an attribute for the categorization process. One measure that has been proposed is cue-validity, which is defined in (24).

**Table:** 

(24)	$\text{cue-validity}\left(C,VA\right):=P\left(C|VA\right)=\frac{P\left(C\wedge VA\right)}{P\left(VA\right)}=\frac{P\left(VA|C\right)\cdot P\left(C\right)}{\overset{n}{\underset{i=1}{\sum }}P\left(VA|C_{i}\right)\cdot P\left(C_{i}\right)}$.

The *C*_*i*_'s in (24) are contrast classes, i.e. siblings of a common superordinate category. One example of such sibling categories is fruit and vegetable. Let us illustrate this definition by an example taken from Schuster (2016). Both fruit and vegetable have similar values in the color attribute, e.g., “green,” “red,” “yellow,” and “orange.” In general, only knowing that an object has the value “green” for this color attribute, the probability to categorize it as a vegetable is high, that is, one has that (24) is high whereas the corresponding cue-validity values for other values of the color attribute are lower and, say, equally probable. Given this fact that one cue-validity value is high, the color attribute should receive a high discriminative value for vegetable because peaks in the probability distribution of attribute values indicate a high discriminative strength for this attribute relative to other contrast classes or categories.

For the definition of diagnosticity, we follow Schuster (2016) and define the diagnosticity of an attribute *A* for a category *C* in terms of the maximum of the cue-validity of each of the *n* values of the attribute. Suppose there are *m* attributes *A*_1_…*A*_*m*_. Let attribute *A*_*i*_, 1 ≤ *i* ≤ *m*, have *n* values *V*_*i*1_…*V*_*in*_. One then first defines *d*(*C, A*) as the maximum of the reversed conditional probabilities of each of the *n* values of *A*.

**Table:** 

(25)	*d*(*C, A*): = *max*(*P*(*C*|*V*_1_*A*), …, (*P*(*C*|*V*_*n*_*A*)) = *max*(cue-validity(*C, V*_1_*A*), …, cue-validity(*C, V*_*n*_*A*)).

Given *d*, diagnosticity of attribute *A*_*i*_ in category *C* is defined as follows.

**Table:** 

(26)	$diag\left(A_{i},C\right):=\frac{d\left(C,A_{i}\right)}{\Sigma_{j=1}^{m}d\left(C,A_{j}\right)}$

One question that needs to be answered is how contrast classes (or categories) are determined. In general, diagnosticity is highly context dependent so that the determination of contrast classes depends on the context. We suggest that the contrast classes are category concepts of the same category. In particular, we suggest that the contrast classes are those category concepts in which the values of attributes are changed that give rise to a correlation, i.e., to a context-dependent default inference in the category concept. For example, in the seaside scenario (2-a) with the lifeguards and the swimmers/trainees there are correlations involving the values of the danger attribute and the value of the location attribute of the theme of the caution event: DANGER.LOCATION = *water* ∧ DANGER.CAUSE = *sharks* ⇒ CAUTION.THEME.LOCATION = *water*. By varying the values of the chains of attributes in the antecedent, one gets the set of contrast classes. Contrast classes, therefore, are in effect category concepts of sort “seaside” in which the danger is located elsewhere, say, on the beach or some other location different from the water and in which the cause of the danger is not sharks. In the scenario in (2-a) the conditional probability for the value “water” of the location attribute is (almost) 1, whereas it is (almost) 0 for other locations of the danger like the beach because the danger is related to sharks.

### 3.6. Typicality

Finally, typicality is defined in terms of the diagnosticity of attributes and similarity of values. A preliminary definition for the typicality of category *C*_*CW*_ with respect to a category *C* is given in (27) (see also Smith et al., 1988; Schuster, 2016).

**Table:** 

(27)	$typicality\left(C,C_{\text{CW}}\right)=\overset{m}{\underset{j=1}{\sum }}diag\left(A_{j},C\right)\overset{n}{\underset{i=1}{\sum }}sim\left(C_{\text{CW}},C|V_{i}A_{j}\right)$.

For each attribute *A*_*j*_ and each value *V*_*i*_ of this attribute, the product of its diagnosticity with the similarity of the value is computed and the sum of these products is taken. Since both the number of pre-activated features and diagnosticity depend on the strength of the context (HC vs. LC), the typicality value is dependent on this distinction, For this reason, the computation of typicality must be adapted to these dependencies. We, therefore, suggest to use (28).

**Table:** 

(28)	$typicality\left(C,C_{\text{CW}}\right)=\frac{\overset{m}{\underset{j=1}{\sum }}diag\left(A_{j},C\right)\overset{n}{\underset{i=1}{\sum }}sim\left(C,C_{\text{CW}}|V_{i}A_{j}\right)}{\overset{m}{\underset{j=1}{\sum }}diag\left(A_{j},C\right)\overset{n}{\underset{i=1}{\sum }}sim\left(C,C|V_{i}A_{j}\right)}$.

(28) reflects the fact that the typicality value for *C* itself is lower in an LC scenario than in an HC scenario. In particular, comparing *C* with itself in the denominator yields the maximal value of typicality in the given context. The nominator then computes the degree of typicality of *C*_*CW*_ relative to *C* in that particular context. Typicality is computed for each feature in *C*_*arg*_, provided the corresponding attribute is defined in *C*_*CW*_. The similarity value of the feature in *C*_*CW*_ is its probability in this category, independently of whether a default inference is licensed or not. Hence, typicality is computed only for features that are context independent or that are default inferences licensed by the context. This accounts for the fact that the N400 amplitude is modulated only by a subset of the admissible features of a category, e.g., those related to achieving a particular goal.

Typicality is computed during the computation of update_*t*_(Σ_*conf*_, Σ_*comp*_, Σ_*disconf*_). In particular, one has that if feature *VA* is checked in the above operation, its typicality is computed. Hence, typicality is computed for all features in *C*_*arg*_, provided the corresponding features are defined for *C*_*CW*_.

### 3.7. The Interplay Between Predictability and Plausibility

The N400 amplitude is a function of both the predictability and the plausibility component. How do the two components contribute to this amplitude? For both components, one has to distinguish between HC and LC scenarios. Let us begin with the predictability component. In an HC scenario, more features are pre-activated than in an LC scenario because there are, in general, more context-dependent default inferences in the former than in the latter. One reason for this difference is correlations between features in *C*_*script*_ and *C*_*arg*_, i.e., context-dependent default inferences. If an HC scenario extends an LC scenario, e.g., by providing an embedding context, one has that the set Σ_*conf*_ ∪ Σ_*comp*_ in the HC scenario is larger than in the LC scenario. As a result, entropy reduction is larger in the former kind of scenario. This difference in context-dependent features yields a higher gain in information for the following kinds of critical words. First, there are CWs for which Σ_*conf*_ is large, i.e., the default inferences in *C*_*arg*_ and *C*_*CW*_ are (almost) the same. This is the case for “swimmers” in (2-a) and it also holds for “Frisbees” in (14). Though the associated category has only a few features in common with that of “paddles,” what counts are the features that are activated in the particular context of (14), yielding *C*_*arg*_, and in this respect the overlap with *C*_*CW*_ is large. A second case is CWs for which most features in *C*_*CW*_ are compatible with those in *C*_*arg*_, i.e., for which Σ_*comp*_ is large. An example is “trainees” in (2-a). Here, the context-dependent features like in_water and afloat can possibly apply to trainees so that they are part of Σ_*comp*_. As a result, there is no difference between “swimmers” and “trainees” at this level. In an LC scenario, the influence of the context on *C*_*arg*_ is weaker in the sense that less context-dependent default inferences are licensed (if any such inferences are licensed at all). As a result, the gain in information is, in general, lower than in an HC scenario.

Let us next turn to the plausibility component. In the predictability component, compatible features lead to a gain in information because they can potentially be verified. This is a consequence of the forward-looking character of this component. By contrast, in the plausibility component it is tested how typical the features in *C*_*CW*_ are relative to the graded structure of *C*_*arg*_, i.e., with respect to the information that is predicted by the underlying category together with the preceding context. Hence, this test is based on information that solely derives from given information. This difference shows up in particular for features that are only compatible and, therefore, for non-best non-anomalous completions. Though they positively contribute to the gain in information, they have a low typicality value. There are two reasons for this. First, in an HC scenario diagnosticity for context-dependent features is high because they are discriminative of a particular role in the scenario. This high value boosts the difference in typicality between a best completion and a non-best completion because for these two CWs the difference in similarity is high. Consider again the scenario (2-a). The discourse-dependent default inference that the theme of the caution event is in water and afloat has a high diagnosticity in the category concept whereas diagnosticity is low for other locations of the danger. These features have a low similarity value for “trainees” so that typicality is low though the gain in information is high. This is in contrast to “swimmers.” For this CW, the similarity values for these features are high. As a result, both the gain in information and the typicality are high for this CW.

Let us next consider an LC scenario. In this kind of scenario, less features are pre-activated compared to a corresponding HC scenario. In particular, most features pre-activated are context independent. Since these features belong to both *C*_*arg*_ and *C*_*CW*_, typicality is high for low cloze words like “trainees” in these scenarios. Hence, the two components show contrary behavior for low cloze words. Whereas, in an LC scenario the gain in matched and compatible features is lower, the degree of typicality is higher because there are less pre-activated context-dependent features with a high diagnosticity value for which the similarity value is low. In an HC scenario, the gain in information is higher, but the degree of typicality is lower. This has the effect that for low cloze CW the N400 amplitude can be the same. The gain at the level of the predictability component in the HC scenario, compared to the LC scenario, is compensated by the lower degree of typicality, again compared to the LC scenario. Consider “trainees” in (2). In the HC scenario, the gain in pre-activated features is higher because (i) more features are pre-activated and (ii) most of these features are compatible with the information in the category concept expressed by this word in this context. However, the features are only compatible, which has the effect that they are not typical or even atypical of the category *C*_trainees_. As an effect, the typicality of *C*_trainees_ relative to *C*_*arg*_ is greater in the LC scenario than in the HC scenario.

If only context-independent features are pre-activated, it does not follow that there are no differences in typicality. Recall that in this case there are no other contrast classes. Hence, there are only minor differences in diagnosticity so that differences in similarity play the major role. By way of example, consider the context-independent feature be_in_danger of the theme of caution events. Without a context, there is no peak in the probability distribution that singles out a particular sort. Yet, the probability that swimmers are in danger is higher than that for trainees.

Let us illustrate our approach by discussing two examples in more detail. We start with the holiday resort scenario in (1). Recall that in this scenario there are relations between categories. Palms and pines are both trees, whereas tulips are flowers, and all three sorts of objects are plants. These categorial relations are also reflected at both the predictability and the plausibility component. Context-dependent default inferences for *C*_*arg*_ include habitat = tropics and height = tall.^12^ Given these context-dependent default inferences, one has for the set of non-disconfirmed features Σ_*conf*_ ∪ Σ_*comp*_ that it is largest for “palms,” followed by that for “pines,” which is followed by that for “tulips.” As a result, entropy reduction is largest for “palms,” followed by that for “pines” and that for “tulips” last. The computation of typicality yields the same ordering. For example, since tulips are small and are from a moderate habitat, the similarity values are those of tulip for those features, which are very low. Let us link this example to the definition of typicality. We use the attribute habitat for illustration. We make the simplifying assumption that this attribute has only two values, “tropics” and “moderate” (see Table 2).

**Table 2:** Similarity values for the three CWs.

	**Palms**	**Pines**	**Tulips**
Tropics	High	Low	Low
Moderate	Low	Low	Low

Remember that the similarity values are defined as the minimum of *P*(*V*HABITAT|*C*_*CW*_) and *P*(*V*HABITAT|*C*_*arg*_). Thus, the value “high” requires that the probability is high in both *C*_*arg*_ and *C*_*CW*_. The value “low” is got if the probability is low in either of the two categories. This is the case whenever a feature is confirmed in one category but not in the other. For “palms,” the value “tropics” is high in both categories and, therefore, has a high similarity value. Since the value “moderate” has a low probability in *C*_*arg*_, the similarity value is low too because for similarity the minimum of the similarity values in *C*_*arg*_ and *C*_pine_ is taken. For “pines,” one gets the following. The value “tropics” has a high probability in *C*_*arg*_ and a low probability in *C*_pine_. Since the minimum is taken for similarity, the similarity value is low. The argument for the value “moderate” is similar with the roles of *C*_*arg*_ and *C*_pine_ switched. Now the probability is low in *C*_*arg*_ and high in *C*_pine_. Again, one gets a low similarity value for this value of the habitat attribute. For “tulips,” the argument is the same as that for “pines.” What about the value of diagnosticity? Recall that contrast classes result by modifying the value of a chain of attributes that license a context-dependent default inference. In the scenario in (1), this is the value “tropical” for the way the hotel should look like. Other values yield different looks. Let us assume that there is only one other value that is “moderate” so that there is only one contrast class. The probability *P*(*C*_*arg*_|tropics) gets a high value in the scenario (1), say 0.9, whereas its value in the contrast class is low, say 0.1. For the value “moderate,” the opposite values can be assumed. One, therefore, has that the cue-validity value for “tropics” is $\frac{0.9}{0.9+0.1}=0.9$ and for “moderate” $\frac{0.1}{0.1+0.9}=0.1$.^13^ As a result, habitat has a high discriminative value and this high value even boosts the differences in typicality between “palms” on the one hand and “pines” and “tulips” on the other hand. When taken together, one has that “palms” has a high typicality value relative to habitat, whereas “pines” and “tulips” get a low value.

Let us next illustrate entropy reduction. For the sake of simplicity, we assume that the frame extensions that are considered are directly related to *C*_*arg*_, i.e. they contain the context-dependent features in this category concept. In the scenario in (1), there are two such features: habitat and height. Crossing these two features with the two values for these attributes assumed above yields four frames: *f*_*tt*_ (habitat = tropics and height = tall), *f*_*ts*_ (habitat = tropics and height = small), *f*_*mt*_ (habitat = moderate and height = tall), and *f*_*ms*_ (habitat = moderate and height = small). The frame *f*_*t*_1__ at *t*_1_ is the category concept for the theme of a planting event that only contains context-independent default inferences. The frame *f*_*t*_2__ at *t*_2_ is the extension of *f*_*t*_1__ that in addition contains the features from Σ_*conf*_ ∪ Σ_*comp*_. For the scenario with the CW “palms,” both features belong to *f*_*t*_2__, i.e., *f*_*t*_2__ = *f*_*tt*_. For the scenario with the CW “pines”, only the height feature belongs to *f*_*t*_2__, i.e., *f*_*t*_2__ = *f*__*t*_ because height = tall is confirmed. Finally, for the scenario with the CW “tulips,” one has *f*_*t*_1__ = *f*_*t*_2__ because all predicted context-independent features are disconfirmed, i.e., prediction error is maximal. Hence, at *t*_1_ all four frames are possible extensions (maximal entropy) whereas at *t*_2_ the extensions depend on which features were not disconfirmed. For “palms,” there are no such extensions because both features were confirmed. By contrast, for “tulips” all four extensions are still possible. In the case of “pines,” there are two extensions because the height feature is confirmed whereas the habitat feature is disconfirmed so that it is not an element of Σ_*conf*_ ∪ Σ_*comp*_.

Let us suppose the following conditional probabilities at *t*_1_: *P*(*f*_HABITAT = *tropics*_|*f*_*t*_1__) = 0.9, *P*(*f*_HABITAT = *moderate*_|*f*_*t*_1__) = 0.1, *P*(*f*_HEIGHT = *tall*_|*f*_*t*_1__) = 0.6, and *P*(*f*_HEIGHT = *small*_|*f*_*t*_1__) = 0.4. Hence, the conditional probabilities for the four extensions are *P*(*f*_*tt*_|*f*_*t*_1__) = 0.54, *P*(*f*_*ts*_|*f*_*t*_1__) = 0.36, *P*(*f*_*mt*_|*f*_*t*_1__) = 0.06, and *P*(*f*_*ms*_|*f*_*t*_1__) = 0.04. Entropy at *t*_1_ is 0.433. If “palms” is encountered, there are no extensions because the frame at *t*_2_ contains both features. Hence, entropy at *t*_2_ is 0 so that entropy reduction is 0.433. If “tulips” is encountered, one has that *f*_*t*_1__ = *f*_*t*_2__ because all predicted context-dependent features are disconfirmed. Hence, entropy remains the same so that there is no reduction in entropy. For “pines,” the situation is different. In this case, there are two frame extensions, adding either habitat= tropics or habitat= moderate. One has *P*(*f*_HABITAT = *tropics*_|*f*__*t*_) = 0.9 and *P*(*f*_HABITAT = *moderate*_|*f*__*t*_) = 0.1. Entropy at *t*_2_ is 0.14. As a result, entropy reduction between *t*_1_ and *t*_2_ is 0.29 if “pines” is encountered.

The second example is the scenario of a birthday party from above and repeated below in (29), which uses prenominal elements.

**Table:** 

(29)	Frank was throwing a birthday party, and he had made the dessert from scratch. After everyone sang, he sliced up some sweet/healthy and tasty cake/veggies that looked delicious.

The context prior to the adjective raises expectations (pre-activates features) that are related to a birthday party, in particular, features that belong to categories expressed by food that is typically served on such an occasion. For our example, we assume that *C*_*arg*_ contains default inferences for the attribute taste with the values “sweet” and “non sweet,” the attribute nutrition_value with the values “healthy” and “non healthy,” and the attribute served_at with values “birthday party” and “non birthday party.”^14^ Let the probabilities be taste = “sweet”: 0.95, taste = “not sweet”: 0.05, nutrition_value = “healthy”: 0.05 and nutrition_value = “non healthy”: 0.95, served_at = “birth party”: 0.98, and served_at = “not birthday party”: 0.02. The values for the attribute served_at reflect the fact that it is known that the context is a birthday party. For diagnosticity, the following assumption is made. Being a birthday, the attributes taste and served_at are more diagnostic (relevant) than the attribute nutrition_value. Hence, the weight on the former two attributes is higher than on the latter one. Let us assume that it is 0.45 for the former two and 0.1 for the latter.^15^

*C*_*arg*_ before prenominal element:

$$\left[\begin{array}{cccc} feature & diag. & sim. & diag.*sim.\\ sweet & 0.45 & 0.95 & 0.4275\\ notsweet & 0.45 & 0.05 & 0.0225\\ healthy & 0.1 & 0.05 & 0.005\\ nothealthy & 0.1 & 0.95 & 0.095\\ served\text{\_}bp & 0.45 & 0.98 & 0.441\\ notserved\text{\_}bp & 0.45 & 0.02 & 0.009 \end{array}\right]$$

Encountering “sweet” confirms these expectations and raises the probability of taste = “sweet” to 1 because it is bottom-up information and it lowers the expectation for nutrition_value = “healthy” to, say, 0.02.^16^ Hence, one gets taste = “sweet”: 1, taste = “not sweet”: 0, nutrition_value = “healthy”: 0.02, and nutrition_value = “not healthy”: 0.98 in *C*_*arg*_.

*C*_*arg*_ after prenominal element “sweet”:

$$\left[\begin{array}{cccc} feature & diag. & sim. & diag.*sim.\\ sweet & 0.45 & 1 & 0.45\\ notsweet & 0.45 & 0 & 0\\ healthy & 0.1 & 0.02 & 0.002\\ nothealthy & 0.1 & 0,98 & 0.098\\ served\text{\_}bp & 0.45 & 0.98 & 0.441\\ notserved\text{\_}bp & 0.45 & 0.02 & 0.009 \end{array}\right]$$

If eventually “cake” is encountered, typicality is high because the probabilities (similarity values) in *C*_*arg*_ are of the same magnitude as those in *C*_*CW*_. By contrast, if “veggies” is encountered instead, typicality is much lower because now the probabilities for all features go in the opposite direction. Whereas “sweet,” ‘non healthy,” and “birthday party,” all have a high probability in *C*_*arg*_, and the probabilities in *C*_*veggies*_ are low.

$$C_{cake}:\left[\begin{array}{cccc} feature & diag. & sim. & diag.*sim.\\ sweet & 0.45 & 0.9 & 0.405\\ notsweet & 0.45 & 0.1 & 0.045\\ healthy & 0.1 & 0.2 & 0.02\\ nothealthy & 0.1 & 0,8 & 0.08\\ served\_bp & 0.45 & 0.98 & 0.441\\ notserved\_bp & 0.45 & 0.02 & 0.009 \end{array}\right]$$

$$C_{veggies}:\left[\begin{array}{cccc} feature & diag. & sim. & diag.*sim.\\ sweet & 0.45 & 0.2 & 0.09\\ notsweet & 0.45 & 0.8 & 0.36\\ healthy & 0.1 & 0.9 & 0.09\\ nothealthy & 0.1 & 0,1 & 0.01\\ served\_bp & 0.45 & 0.02 & 0.009\\ notserved\_bp & 0.45 & 0.98 & 0.441 \end{array}\right]$$

If instead of “sweet” “healthy” is encountered, this raises the probability of this feature, i.e., of nutrition_value = “healthy,” to 1 because it is bottom-up information and it lowers the probability for the feature “sweet” due to the correlation between the two features. Let us assume that the values for taste are updated to taste = “sweet”: 0.4 and taste = “not sweet”: 0.6.

*C*_*arg*_ after prenominal element “healthy”:

$$\left[\begin{array}{cccc} feature & diag. & sim. & diag.*sim.\\ sweet & 0.45 & 0.4 & 0.18\\ notsweet & 0.45 & 0.6 & 0.27\\ healthy & 0.1 & 1 & 0.1\\ nothealthy & 0.1 & 0 & 0\\ served\text{\_}bp & 0.45 & 0.95 & 0.4275\\ notserved\text{\_}bp & 0.45 & 0.05 & 0.0225 \end{array}\right]$$

This has the effect that the typicality for “cake” is lowered (compared to encountering “sweet”) and that the typicality of “veggies” is raised. However, one also has to consider diagnosticity. As already said above, being a birthday, the attributes taste and served_at are more diagnostic (relevant) than the attribute nutrition_value. Hence, the weight on the former two attributes is higher than on the latter one.

**Figure 1:**
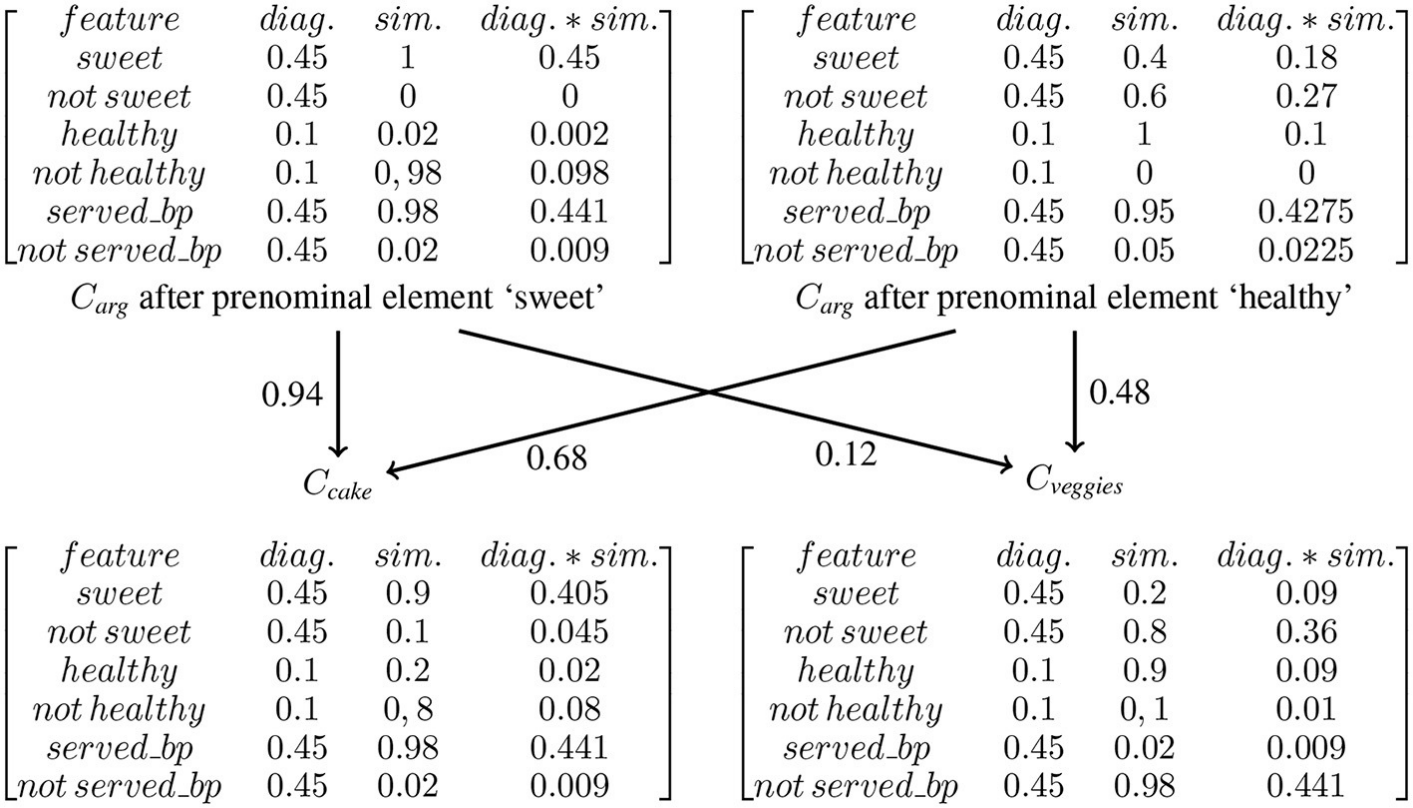
Overview of the diagnosticity, similarity, and typicality values for the four examples in (29). The arrows are labeled by the typicality values.

Using (27)^17^, one gets the following typicality values where *C*_*x*_ is *C*_*arg*_ after prenominal element *x*: *typicality*(*C*_*sweet*_, *C*_*cake*_) = 0.937, *typicality*(*C*_*healthy*_, *C*_*cake*_) = 0.6815, *typicality*(*C*_*healthy*_, *C*_*veggies*_) = 0.4815, and *typicality*(*C*_*sweet*_, *C*_*veggies*_) = 0.12. See Figure 1 for an overview of the results for this example.

For the predictability component, one gets the following. For “sweet (and tasty) cake,” all three default inferences are confirmed, while “sweet (and tasty) veggies” disconfirms all three inferences. The interesting cases are “healthy (and tasty) cake” and “healthy (and tasty) veggies.” Since encountering the prenominal element “healthy” leads to a change for the feature “sweet,” now “not sweet” is expected, “sweet (and tasty) cake” confirms only one default inference in *C*_*arg*_, whereas “healthy (and tasty) veggies” confirms two. However, one has to bear in mind that we have restricted the examples to three attributes. If more attributes are considered, e.g., the way the food is prepared, which ingredients are used, etc, “healthy (and tasty) cakes” will confirm more inferences.

The last two examples show how the two components underlying N400 activity interact with each other in our approach. In particular, they show how the two components depend on world knowledge and how features that are given by prenominal elements before the CW is encountered modulate the N400 amplitude.

### 3.8. The N400 and Schema-Based Knowledge

Our approach assumes that the N400 amplitude is sensitive to both the number of disconfirmed features and the typicality of *C*_*CW*_ relative to *C*_*arg*_. However, the examples below in (30) seem to be counterexamples to our approach.

**Table:** 

(30)	a.	A huge blizzard swept through town last night. My kids ended up getting the day off from school. They spent the whole day outside building a big snowman / jacket / towel in the front yard.
	b.	The prescription for the mental disorder was written by the psychiatrist / schizophrenic / guard / pill / fence ….

Though both “jacket” and “towel” in (30-a) taken from Metusalem et al. (2012) share few features with the best completion “snowman” and both words are highly atypical given the partial event structure built up preceding the CW in the target sentence, “jacket” elicited a reduced N400 amplitude compared to “towel.” However, this attenuation only occurred when the target sentence was embedded in the wider context given in (30-a) and not when it was used in isolation. A similar argument holds for (30-b) taken from Vega-Mendoza et al. (2021), which is a replication study of Paczynski and Kuperberg (2012). The authors found the following pattern of N400 amplitude per condition: plausible control (psychiatrist) < animate-related (schizophrenic) < animate-unrelated (guard) < inanimate-related (pill) < inanimate-unrelated (fence). Furthermore, this pattern followed the pattern of plausibility judgments with larger N400 found for increasingly implausible conditions.^18^

So far, we assumed that *C*_*arg*_ is related to one particular argument position, e.g., the theme of the event of sort “build” in (30-a) or the actor of the writing in (30-b). We take the examples in (30) as evidence that this need not always be the case. Rather, instead of a unique *C*_*arg*_ several such category concepts can be determined by the scenario that is described by the context. This raises the question of which arguments or objects can be targeted by category concepts that are related to other arguments or objects. Recall that *C*_*arg*_ contains pre-activated features. One kind of attributes is the properties of the expected object, e.g., whether it is sweet (taste), is healthy (nutritional_value), or is in water (location). The second kind of attribute relates the expected object to other objects. For example, the prescription can be related to its recipient (e.g., a schizophrenic) and the prescribed medicine (e.g., some kind of pills). Let us call such attributes *object-related*. Now the thesis is that *C*_*arg*_ can be related to an attribute that is object-related. On this generalized, view a *C*_*arg*_ is related to an extension of the information (frame) about an object that is going to be introduced or that has already been introduced into the scenario or the event structure. In the previously discussed examples, *C*_*arg*_ is related to the event denoted by the verb and the object itself has not yet been introduced. The problematic cases in (30) are instances in which *C*_*arg*_ is linked to an object that has already been introduced. In (30-a), there is a *C*_*arg*_ that is linked to the clothes of the children and in (30-b) there is a *C*_*arg*_ that is linked to the recipient of the prescription and another one that is linked to the medicine prescribed. If several *C*_*arg*_ can be activated, the question has to be answered how their typicality can be computed. Recall that each *C*_*arg*_ is related to a particular argument position, which, in turn, expresses a particular thematic role that links the event denoted by the verb to the object denoted by the argument. One possibility, therefore, is to make this dependency explicit by relating each pre-activated feature to a particular thematic role. Thus, if the feature π is an element of a *C*_*arg*_, it is replaced by *tr* • π for *tr* the thematic role and • denoting the operation of chain concatenation. Hence, *tr* is an attribute. How is the diagnosticity of these attributes defined? We hypothesize that the diagnosticity is the expectation that CW provides information about this role. This expectation is highest for thematic roles that are related to undischarged arguments. For example, in (30-a) information about the theme is most expected whereas in (30-b) it is information about the actor. For the values of the thematic role attributes *tr*, diagnosticity is computed as defined above in section 3.6. The typicality value of a chain *tr* • π is computed by multiplying the diagnosticity of *tr* with the typicality of π. For the latter value, this means that selection restrictions imposed by the verb have to be taken into account, in particular, the animacy constraints. For example, for (30-b), this has the effect that features related to animate objects have higher typicality than features that are related to inanimate objects. Hence, the diagnosticity (expectancy) of *tr* and the animacy constraint interact with each other. For example, one has that a feature for an undischarged argument that is related to an object that satisfies the animacy constraint has higher typicality than a feature for a discharged argument that is related to an object that fails to satisfy the animacy constraint because in the latter case diagnosticity for *tr* is lower and the similarity value will be very low due to the violation. This is the case for “fence” in (30-b). For features for discharged arguments that satisfy the animacy constraint and that have both a high diagnosticity and a high similarity value, the overall typicality can be high even if the diagnosticity for *tr* is lower than in the case of an undischarged argument. This is the case for “schizophrenic” in (30-b). It is related to the recipient of the prescription (high diagnosticity) and due to the information that it is for a mental disorder this sort of recipient has a high similarity. The difference between “schizophrenic” and “guard” is that the latter has a low similarity value both for the actor role and for the recipient role in the frame related to the prescription.

## 4. Outlook and Conclusions

The theoretical account of a hybrid view of the N400 developed in this study has so far not been empirically tested. An important question, therefore, is to design experimental tests that provide evidence for or against it. The first, and important, strategy is related to the theoretical dimension. Given that we interpret N400 in terms of two operations, it must be possible to define a (monotone) function taking these two operations as arguments that correlates with the N400 amplitude (see Werning et al., 2019 for a similar strategy in a different theoretical setting). At the empirical dimension, two interesting strategies are the following. Our approach assumes that predictions are related to particular concepts or category concepts. As already mentioned above, Wang et al. (2020) have shown, using RSA in combination with EEG/MEG, that animacy features related to an argument position of a verb are pre-activated upon processing the verb before the argument is encountered. Such predictions should not be restricted to animacy features but should also include finer-grained categories that are related to other selection restrictions or the context. For example, upon encountering “cautioned” in (2-a) not only animacy features but also features that are related to the concept “danger” should be activated (see also Wang et al., 2020 for a similar argument in relation to other categories). A more general question is whether it is possible to detect differences between animacy, other selection restrictions, constraints imposed by the event structure, and constraints imposed by the scenario (script knowledge). A second empirical test is related to revisions that are triggered by mismatching prenominal elements. According to our approach, such revisions should not index the plausibility of the resulting event structure but a shift in the probability distribution of which kinds of objects are expected. This should result in a different set of features that are pre-activated. Hence, an interesting question is to combine the methods used in Wang et al. (2020) and Fleur et al. (2020). On a mismatching prenominal element, the activation pattern (measured using RSA with EEG/MEG) should change.

On a more theoretical side, one has that the definitions of similarity, diagnosticity, and typicality given here are only one option among others. What are alternatives and how can the choice between them be empirically tested? Furthermore, the approach must be extended to additional data. Of particular interest are data that seem to provide evidence that plausibility does play no role in the modulation of the N400 amplitude. An example is the results from Delogu et al. (2019).

## Data Availability Statement

The original contributions presented in the study are included in the article, further inquiries can be directed to the corresponding author.

## Author Contributions

All authors listed have made a substantial, direct and intellectual contribution to the work, and approved it for publication.

## Funding

This article grew out of work of the authors in the CRC 991 “The Structure of Representations in Language, Cognition, and Science,” funded by the German Science Foundation (DFG).

## Conflict of Interest

The authors declare that the research was conducted in the absence of any commercial or financial relationships that could be construed as a potential conflict of interest.

## Publisher's Note

All claims expressed in this article are solely those of the authors and do not necessarily represent those of their affiliated organizations, or those of the publisher, the editors and the reviewers. Any product that may be evaluated in this article, or claim that may be made by its manufacturer, is not guaranteed or endorsed by the publisher.

## Footnotes

^1^More specifically, the authors still observed a slightly larger N400 for “slow” compared to “quick.” However, as noted by the authors, inspecting the grand average ERPs clearly showed that a substantial part of the N400 effect elicited by “slow” was eliminated if the target sentence was presented without the embedding context. This was confirmed by a joint ANOVA on mean amplitude in the 300 to 500 msec latency range (van Berkum et al., 1999, p.661).

^2^The following paragraphs owe a lot to comments from our editor Gina Kuperberg.

^3^We are indebted to one reviewer for this observation.

^4^The authors suggest that this was the first time point at which comprehenders were able to infer the full high-level event structure (e.g., “agent cautioned animate noun”) and that they used this structure to predict animacy features of the (upcoming) theme (Wang et al., 2020, 3289f.).

^5^The following abbreviations are used: nv: no violation; av: animacy violation; sv: semantic violation. By a semantic violation Szewczyk and Schriefers (2011) understand any violation of a non-animacy selection restriction or a contextual constraint.

^6^This is also noted by Boudewyn et al. (2015) who take their results as showing that the occurrence of an adjective denoting a feature that is atypical of the objects denoted by the expected noun leads a comprehender to dynamically adjust her expectations in such a way that the noun no longer receives the same level of facilitation as in the case of the occurrence of an adjective that denotes a typical feature, and that the presence of a local consistent feature (e.g., healthy) can raise expectations for a noun that denotes objects for which this feature is typical (e.g., veggies).

^7^This study re-analyzed data from the large scale replication study Nieuwland et al. (2018), which is based on the data in DeLong et al. (2005).

^8^It is important to note that the authors underline the exploratory character of the analysis of prediction revision. In particular, the ERP effect associated with revised constraint, i.e., next-word entropy, reached the traditional level of statistical significance only in a subset of the analyses performed by the authors.

^9^We do not assume that a low-constraining context always only pre-activates context-independent features. This is the case only if a comprehender interprets the sentence in a literal way. However, a comprehender may also apply background knowledge or information that is given by the non-linguistic context. Consider the following example: “John is drinking a glass of ….” If this sentence is given in a study, it will be low-constraining because many beverages will be mentioned. However, if “John” denotes a particular person who is a strict anti-alcoholic, a comprehender who knows John will pre-activate a category concept with features that only apply to non-alcoholic beverages. An example of a non-linguistic factor is information in spoken language about the age of the speaker. If the sentence “I always read the newspaper before I leave” is uttered by the voice of a young child, an N400 is elicited on the CW, van Berkum et al. (2008). If, by contrast, this sentence is read in silence by a comprehender, this will not be the case.

^10^A more detailed formalization can be found in Naumann and Petersen (2021).

^11^The determination of *r* is an empirical question and will in general also depend on the context.

^12^For simplicity, we do not state the complete chains of attributes but only the last attribute together with the value.

^13^For the sake of simplicity it is assumed that the probabilities of the two contrast classes are the same. Furthermore, the attribute habitat is assumed to form a domain of its own so that in the denominator of (26) the sum is only over this attribute.

^14^For the sake of simplicity, we leave out the prenominal element “tasty.”

^15^Diagnosticity is determined for the attributes of *C*_*arg*_ because it is the typicality of *C*_*CW*_ relative to *C*_*arg*_ that is computed. Below we include these diagnosticity values in the frames of *C*_*cake*_ and *C*_*veggies*_ to ease understanding of how the typicality values are computed.

^16^Using standard Bayesian update, the new probability of a feature *f* given the feature *f*_*pe*_ associated with the prenominal element *pe* is given by *p*(*f*|*f*_*pe*_). In the given case, one has *f*_*pe*_= taste = “sweet” and an example of *f* is nutrition_value = “healthy.”

^17^We use (27) and not (28) because we do not compare HC and LC scenarios.

^18^Two caveats are in order. First, this pattern was found only when participants performed a plausibility judgment task but not when they only passively read the sentences. In this case, the authors found that inanimate nouns elicited N400 effects compared to the control nouns and compared to animate nouns whereas the N400 amplitudes for the animate nouns did not differ from the control nouns. Second, in the original study Paczynski and Kuperberg (2012) the authors found (a) an interaction wherein related words elicited smaller N400 amplitudes than unrelated words when these words were animate, but not when they were inanimate and (b) animate-related words like “schizophrenic” did not elicit a reliable N400 effect compared to control words. For a discussion of the differences in the methodological design of the two studies, see Vega-Mendoza et al. (2021).
